# Supplementary material for: Elucidating the genetic basis of biomass accumulation and radiation use efficiency in spring wheat and its role in yield potential
Source: Plant Biotechnol J. 2019 Jan 15;17(7):1276–88. doi: 10.1111/pbi.13052 (PMC6576103; doi:10.1111/pbi.13052)
Supplement: Supplementary file 1 — Figure S1 Histograms of the distribution of the phenotypic values of plant height, anthesis date (DTA) and days to maturity (DTM). Figure S2 Boxplots of the best linear estimated predictions (BLUEs) of the main traits measured in HiBAP during 2 years of evaluation (Y16&Y17). Figure S3 Linkage Disequilibrium Decay depicted as a scatter plot of pairwise SNP LD (R 2) and pairwise physical distance across the hexaploid wheat genome. Figure S4 GWAS results using 9267 SNPs markers in HiBAP for yield traits based on BLUEs means obtained from the combined analysis from Y16 and Y17. Figure S5 Venn diagram exhibiting the number of total and common MTA's detected for different traits. Table S1 Growing conditions and main agronomical characteristics of the trial grown for 2 years in northeast Mexico under full irrigation conditions. Table S2 Phenotypic correlations among the 31 traits presented in this study. Table S3 Genetic correlations among the 31 traits presented in this study. Table S4 The distribution of the 35K Axiom Wheat Breeders Array loci on the Refseq1.0 Chinese Spring wheat physical map. Table S5 The distribution of 35K Axiom array SNPs that were polymorphic in the HiBAP panel. Table S6 Linkage Disequilibrium/Decay statistics for the HiBAP panel. Table S7 Summary of GWAS results from the trial evaluated during 2 years in northeast Mexico under full irrigation conditions. Table S8 List of selected candidate genes found for the evaluated traits using GWA mapping. [file PBI-17-1276-s001.docx]

Plant Biotechnology Journal Supporting Information

Elucidating the genetic basis of biomass accumulation and radiation use efficiency in spring wheat and its role in yield potential

Gemma Molero^1*^, Ryan Joynson^2*^, Francisco J. Pinera-Chavez^1^, Laura-Jayne Gardiner^2^, Carolina Rivera-Amado^1^, Anthony Hall^†2^ and Matthew P. Reynolds^†1^

^1^ Global Wheat Program, International Maize and Wheat Improvement Centre (CIMMYT), Texcoco, Mexico

^2^The Earlham Institute, Norwich, UK

^*^**both authors have contributed equally to the paper**

The following Supporting Information is available for this article:

**Table S1.** Growing conditions and main agronomical characteristics of the trial grown for two years in northeast Mexico under full irrigation conditions.

**Table S2.** Phenotypic correlations among the 31 traits presented in this study. Bold numbers indicate that the correlation is significant at least at *P*<0.05.

**Table S3.** Genetic correlations among the 31 traits presented in this study. Bold numbers indicate that the correlation is

**Table S4.** The distribution of the 35K Axiom Wheat Breeders Array loci on the Refseq1.0 Chinese Spring wheat physical map.

**Table S5.** The distribution of 35K Axiom array SNPs that were polymorphic in the HiBAP panel.

**Table S6.** Linkage Disequilibrium/Decay statistics for the HiBAP panel.

**Table S7.** Summary of GWAS results. Common SNPs are indicated by the same colour

**Table S8**. List of selected candidate genes found for the evaluated traits using GWA mapping.

**Figure S1**. Histograms of the distribution of the phenotypic values of plant height, anthesis date (DTA) and days to maturity (DTM).

**Figure S2.** Boxplots of the best linear estimated predictions (BLUEs) of the main traits measured in HiBAP during two years of evaluation (Y16&Y17).

**Figure S3.** Linkage Disequilibrium Decay depicted as a scatter plot of pairwise SNP LD (R^2^) and pairwise physical distance across the hexaploid wheat genome.

**Figure S4.** GWAS results using 21,708 SNPs markers in HiBAP for yield traits based on BLUEs means obtained from the combined analysis from Y16 and Y17.

**Figure S5**. Venn diagram exhibiting the number of total and common MTA’s detected for different traits.

Table S1. Growing conditions and main agronomical characteristics of the trial grown for two years in northeast Mexico under full irrigation conditions. Temperature, relative humidity, solar radiation and evapotranspiration (ETo) are monthly averages. Rain is monthly total. Year 1 the experiment was sown under dry soil whereas year 2 the experiment was sown under moisture.

| **Crop Cycle** | **Sowing** | **Emergence** | **Harvest** | **DTA** | **DTM** | **Fertilization N-P-K** | **Irrigations** | **Temp Mean (Min, Max)** | **RH** | **Radiation** | **Rain** | **ETo** |
| --- | --- | --- | --- | --- | --- | --- | --- | --- | --- | --- | --- | --- |
|  |  |  |  |  |  | kg ha^-1^ | n | ^o^C | % | MJ m^-2^ day^-1^ | mm | mm |
| 2015/2016 | 24-Nov-15 | 7-Dec-15 | 16/20-May-16 | 79 | 118 | 250-50-00 | 5 | 16.7 (8.7, 26.6) | 69.3 | 17.3 | 22.9 | 3.6 |
| 2016/2017 | 23-Nov-16 | 30-Nov-16 | 26-Apr-17 | 74 | 112 | 250-50-00 | 4 | 17.3 (10.1, 26.4) | 72.4 | 15.0 | 42.5 | 3.4 |

**Table S2.** Phenotypic correlations among the 31 traits presented in this study. Bold numbers indicate that the correlation is significant at least at *P*<0.05.

|  | **1** | **2** | **3** | **4** | **5** | **6** | **7** | **8** | **9** | **10** | **11** | **12** | **13** | **14** | **15** | **16** |
| --- | --- | --- | --- | --- | --- | --- | --- | --- | --- | --- | --- | --- | --- | --- | --- | --- |
| **1. YLD** | 1 |  |  |  |  |  |  |  |  |  |  |  |  |  |  |  |
| **2. Height** | -0.007 | 1 |  |  |  |  |  |  |  |  |  |  |  |  |  |  |
| **3. Plm2** | **-0.363** | **-0.164** | 1 |  |  |  |  |  |  |  |  |  |  |  |  |  |
| **4. Stm2_E40** | **-0.298** | **-0.191** | **0.409** | 1 |  |  |  |  |  |  |  |  |  |  |  |  |
| **5. Stm2_InB** | **-0.318** | **-0.178** | **0.371** | **0.723** | 1 |  |  |  |  |  |  |  |  |  |  |  |
| **6. Stm2_A7** | **-0.192** | **-0.339** | **0.289** | **0.475** | **0.613** | 1 |  |  |  |  |  |  |  |  |  |  |
| **7. DTInB** | **-0.201** | -0.018 | **0.268** | **0.39** | **0.324** | 0.127 | 1 |  |  |  |  |  |  |  |  |  |
| **8. DTA** | -0.148 | 0.028 | **0.314** | **0.321** | **0.265** | 0.035 | **0.902** | 1 |  |  |  |  |  |  |  |  |
| **9. DTM** | -0.154 | -0.079 | **0.342** | **0.279** | **0.298** | 0.104 | **0.778** | **0.867** | 1 |  |  |  |  |  |  |  |
| **10. RSGP** | **0.186** | 0.081 | -0.037 | **-0.275** | **-0.251** | **-0.236** | **-0.571** | **-0.166** | **-0.205** | 1 |  |  |  |  |  |  |
| **11. PGF** | 0.095 | -0.128 | **-0.169** | **-0.255** | -0.128 | 0.062 | **-0.732** | **-0.803** | **-0.4** | 0.068 | 1 |  |  |  |  |  |
| **12. HI** | **0.398** | **-0.386** | **-0.281** | -0.067 | **-0.18** | 0.05 | **-0.321** | **-0.431** | **-0.442** | -0.055 | **0.268** | 1 |  |  |  |  |
| **13. TGW** | **0.323** | **0.485** | **-0.537** | **-0.449** | **-0.466** | **-0.446** | **-0.171** | **-0.168** | **-0.273** | 0.088 | -0.009 | -0.022 | 1 |  |  |  |
| **14. GM2** | **0.332** | **-0.496** | **0.282** | **0.223** | **0.225** | **0.302** | -0.046 | -0.015 | 0.095 | 0.059 | 0.143 | **0.304** | **-0.778** | 1 |  |  |
| **15. SM2** | -0.006 | **-0.4** | **0.291** | **0.523** | **0.573** | **0.625** | 0.002 | -0.054 | 0.057 | -0.094 | 0.144 | -0.078 | **-0.484** | **0.473** | 1 |  |
| **16. GWSP** | **0.454** | **0.345** | **-0.441** | **-0.6** | **-0.636** | **-0.618** | -0.059 | 0.005 | -0.099 | 0.144 | -0.111 | **0.243** | **0.59** | **-0.283** | **-0.862** | 1 |
| **17. GSP** | **0.23** | -0.059 | -0.019 | **-0.311** | **-0.333** | **-0.311** | 0.073 | 0.155 | 0.143 | 0.106 | -0.099 | **0.283** | **-0.252** | **0.424** | **-0.568** | **0.617** |
| **18. SPKLSP** | -0.027 | 0.151 | 0.122 | 0.035 | 0.011 | -0.153 | **0.557** | **0.624** | **0.61** | -0.113 | **-0.414** | **-0.232** | **-0.229** | **0.161** | **-0.197** | **0.187** |
| **19. InfSPKLSP** | -0.017 | 0.145 | 0.149 | 0.146 | 0.108 | 0.094 | **0.179** | **0.244** | **0.258** | 0.038 | -0.14 | **-0.18** | -0.156 | 0.112 | 0.146 | -0.132 |
| **20. Spike** | 0.157 | **0.417** | -0.032 | **-0.21** | **-0.271** | **-0.398** | **0.17** | **0.287** | **0.262** | 0.141 | **-0.218** | -0.108 | **0.186** | -0.112 | **-0.485** | **0.502** |
| **21. BME40** | -0.014 | **0.208** | 0.093 | **0.389** | **0.208** | 0.083 | **-0.258** | **-0.26** | **-0.29** | 0.098 | 0.149 | 0.041 | 0.137 | -0.122 | 0.073 | -0.091 |
| **22. BMInB** | -0.119 | **0.204** | 0.09 | **0.207** | **0.38** | 0.143 | **0.384** | **0.341** | **0.284** | **-0.26** | **-0.26** | **-0.376** | 0.099 | **-0.2** | -0.043 | 0.001 |
| **23. BMA7** | 0.097 | **0.265** | -0.052 | **-0.165** | 0.018 | **0.323** | **-0.22** | **-0.172** | **-0.204** | **0.19** | 0.08 | **-0.163** | **0.165** | -0.084 | 0.022 | 0.039 |
| **24. BMPM** | **0.561** | **0.365** | **-0.161** | **-0.216** | -0.134 | **-0.213** | 0.11 | **0.236** | **0.228** | **0.174** | -0.154 | **-0.462** | **0.344** | 0.006 | 0.06 | **0.256** |
| **25. RUE_E40InB** | 0.145 | 0.061 | **-0.245** | **-0.320** | 0.060 | 0.036 | **-0.615** | **-0.559** | **-0.437** | **0.323** | **0.524** | 0.006 | **0.162** | -0.008 | 0.023 | 0.057 |
| **26. RUE_InBA7** | 0.113 | 0.044 | -0.051 | -0.131 | -0.097 | **0.417** | **-0.188** | **-0.256** | **-0.253** | -0.044 | **0.170** | 0.119 | 0.028 | 0.070 | 0.114 | -0.049 |
| **27. RUE_GF** | **0.533** | 0.139 | **-0.213** | **-0.204** | **-0.221** | **-0.389** | 0.009 | 0.090 | 0.000 | **0.170** | **-0.170** | **-0.219** | **0.274** | 0.068 | 0.122 | **0.198** |
| **28. RUET** | **0.609** | **0.265** | **-0.398** | **-0.429** | **-0.297** | **-0.198** | **-0.360** | **-0.299** | **-0.360** | **0.284** | 0.121 | -0.160 | **0.453** | -0.024 | 0.058 | **0.281** |
| **29. LIE40** | 0.008 | **0.27** | 0.09 | 0.066 | 0.014 | -0.033 | -0.004 | -0.024 | -0.11 | -0.043 | -0.075 | -0.062 | **0.292** | **-0.278** | -0.152 | 0.144 |
| **30. LIInB** | -0.019 | **0.253** | **0.205** | **0.23** | **0.177** | -0.071 | **0.706** | **0.721** | **0.587** | **-0.237** | **-0.627** | **-0.374** | 0.019 | -0.093 | -0.12 | 0.106 |
| **31. LIA7** | -0.046 | **0.254** | 0.151 | 0.141 | 0.155 | -0.001 | **0.445** | **0.533** | **0.42** | -0.005 | **-0.474** | **-0.378** | 0.118 | **-0.195** | -0.064 | 0.04 |

|  | **17** | **18** | **19** | **20** | **21** | **22** | **23** | **24** | **25** | **26** | **27** | **28** | **29** | **30** | **31** |
| --- | --- | --- | --- | --- | --- | --- | --- | --- | --- | --- | --- | --- | --- | --- | --- |
| **17. GWSP** | 1 |  |  |  |  |  |  |  |  |  |  |  |  |  |  |
| **18. SPKLSP** | **0.422** | 1 |  |  |  |  |  |  |  |  |  |  |  |  |  |
| **19. InfSPKLSP** | -0.04 | **0.397** | 1 |  |  |  |  |  |  |  |  |  |  |  |  |
| **20. Spike** | **0.393** | **0.579** | 0.131 | 1 |  |  |  |  |  |  |  |  |  |  |  |
| **21. BME40** | **-0.261** | **-0.299** | -0.025 | -0.122 | 1 |  |  |  |  |  |  |  |  |  |  |
| **22. BMInB** | -0.117 | **0.226** | 0.094 | 0.027 | **0.247** | 1 |  |  |  |  |  |  |  |  |  |
| **23. BMA7** | -0.113 | -0.048 | 0.106 | 0.102 | 0.153 | **0.303** | 1 |  |  |  |  |  |  |  |  |
| **24. BMPM** | -0.009 | **0.211** | 0.148 | **0.237** | -0.044 | **0.219** | **0.25** | 1 |  |  |  |  |  |  |  |
| **25. RUE_E40InB** | -0.075 | **-0.297** | -0.131 | **-0.181** | 0.159 | **0.268** | **0.427** | 0.143 | 1 |  |  |  |  |  |  |
| **26. RUE_InBA7** | -0.074 | -0.147 | 0.009 | -0.037 | -0.038 | **-0.204** | **0.721** | 0.027 | 0.051 | 1 |  |  |  |  |  |
| **27. RUE_GF** | -0.013 | 0.009 | -0.015 | 0.022 | -0.108 | -0.078 | **-0.248** | **0.742** | 0.046 | **-0.306** | 1 |  |  |  |  |
| **28. RUET** | -0.083 | **-0.191** | -0.081 | -0.020 | -0.008 | -0.005 | **0.378** | **0.773** | **0.451** | **0.221** | **0.688** | 1 |  |  |  |
| **29. LIE40** | -0.126 | 0.008 | -0.008 | **0.17** | **0.355** | **0.347** | **0.167** | 0.101 | 0.057 | -0.008 | -0.009 | 0.046 | 1 |  |  |
| **30. LIInB** | 0.103 | **0.479** | **0.161** | **0.376** | -0.059 | **0.374** | 0.003 | **0.331** | **-0.398** | -0.122 | 0.145 | -0.053 | **0.175** | 1 |  |
| **31. LIA7** | -0.068 | **0.291** | 0.133 | **0.329** | 0.019 | **0.27** | 0.15 | **0.291** | **-0.262** | -0.006 | 0.082 | 0.008 | **0.218** | **0.593** | 1 |

**Table S3.** Genetic correlations among the 31 traits presented in this study. Bold numbers indicate that the correlation is

|  | **1** | **2** | **3** | **4** | **5** | **6** | **7** | **8** | **9** | **10** | **11** | **12** | **13** | **14** | **15** | **16** |
| --- | --- | --- | --- | --- | --- | --- | --- | --- | --- | --- | --- | --- | --- | --- | --- | --- |
| **1. YLD** | 1 |  |  |  |  |  |  |  |  |  |  |  |  |  |  |  |
| **2. Height** | -0.011 | 1 |  |  |  |  |  |  |  |  |  |  |  |  |  |  |
| **3. Plm2** | **-0.771** | **-0.3** | 1 |  |  |  |  |  |  |  |  |  |  |  |  |  |
| **4. Stm2_E40** | **-0.504** | **-0.278** | **0.876** | 1 |  |  |  |  |  |  |  |  |  |  |  |  |
| **5. Stm2_InB** | **-0.503** | **-0.243** | **0.744** | **0.99** | 1 |  |  |  |  |  |  |  |  |  |  |  |
| **6. Stm2_A7** | **-0.315** | **-0.48** | **0.602** | **0.785** | **0.951** | 1 |  |  |  |  |  |  |  |  |  |  |
| **7. DTInB** | **-0.284** | -0.022 | **0.482** | **0.557** | **0.433** | **0.176** | 1 |  |  |  |  |  |  |  |  |  |
| **8. DTA** | **-0.204** | 0.033 | **0.551** | **0.448** | **0.347** | 0.048 | **0.99** | 1 |  |  |  |  |  |  |  |  |
| **9. DTM** | **-0.215** | -0.096 | **0.608** | **0.394** | **0.394** | 0.143 | **0.922** | **0.99** | 1 |  |  |  |  |  |  |  |
| **10. RSGP** | **0.355** | 0.133 | -0.09 | **-0.53** | **-0.452** | **-0.441** | **-0.923** | **-0.262** | **-0.328** | 1 |  |  |  |  |  |  |
| **11. PGF** | 0.146 | **-0.169** | **-0.329** | **-0.395** | **-0.185** | 0.094 | **-0.953** | **-0.99** | **-0.515** | 0.12 | 1 |  |  |  |  |  |
| **12. HI** | **0.601** | **-0.503** | **-0.538** | -0.102 | **-0.257** | 0.074 | **-0.411** | **-0.539** | **-0.559** | -0.094 | **0.372** | 1 |  |  |  |  |
| **13. TGW** | **0.43** | **0.558** | **-0.908** | **-0.604** | **-0.587** | **-0.582** | **-0.192** | **-0.185** | **-0.305** | 0.133 | -0.011 | -0.026 | 1 |  |  |  |
| **14. GM2** | **0.469** | **-0.606** | **0.506** | **0.319** | **0.301** | **0.419** | -0.056 | -0.018 | 0.113 | 0.095 | **0.186** | **0.388** | **-0.877** | 1 |  |  |
| **15. SM2** | -0.009 | **-0.509** | **0.544** | **0.778** | **0.798** | **0.903** | 0.003 | -0.066 | 0.071 | -0.158 | **0.196** | -0.103 | **-0.568** | **0.589** | 1 |  |
| **16. GWSP** | **0.646** | **0.424** | **-0.796** | **-0.862** | **-0.856** | **-0.862** | -0.071 | 0.006 | -0.118 | **0.234** | -0.146 | **0.312** | **0.668** | **-0.341** | **-0.99** | 1 |
| **17. GSP** | **0.34** | -0.075 | -0.036 | **-0.464** | **-0.465** | **-0.451** | 0.091 | **0.19** | **0.177** | **0.178** | -0.134 | **0.377** | **-0.297** | **0.529** | **-0.74** | **0.776** |
| **18. SPKLSP** | -0.04 | **0.192** | **0.228** | 0.052 | 0.015 | **-0.22** | **0.694** | **0.759** | **0.751** | **-0.189** | **-0.56** | **-0.308** | **-0.268** | **0.2** | **-0.255** | **0.234** |
| **19. InfSPKLSP** | -0.031 | **0.231** | **0.349** | **0.271** | **0.189** | **0.17** | **0.279** | **0.372** | **0.398** | 0.08 | **-0.237** | **-0.299** | **-0.23** | **0.174** | **0.237** | **-0.207** |
| **20. Spike** | **0.221** | **0.505** | -0.057 | **-0.298** | **-0.359** | **-0.547** | **0.202** | **0.333** | **0.308** | **0.225** | **-0.281** | -0.137 | **0.207** | -0.133 | **-0.6** | **0.599** |
| **21. BME40** | -0.037 | **0.473** | **0.312** | **0.99** | **0.52** | **0.216** | **-0.577** | **-0.568** | **-0.641** | **0.294** | **0.362** | 0.098 | **0.289** | **-0.273** | **0.169** | **-0.205** |
| **22. BMInB** | **-0.263** | **0.389** | **0.251** | **0.461** | **0.794** | **0.309** | **0.718** | **0.623** | **0.527** | **-0.656** | **-0.528** | **-0.752** | **0.174** | **-0.373** | -0.085 | 0.002 |
| **23. BMA7** | **0.178** | **0.42** | -0.12 | **-0.305** | 0.032 | **0.58** | **-0.342** | **-0.261** | **-0.313** | **0.398** | 0.134 | **-0.27** | **0.241** | -0.13 | 0.035 | 0.06 |
| **24. BMPM** | **0.99** | **0.634** | **-0.41** | **-0.439** | **-0.255** | **-0.42** | **0.186** | **0.393** | **0.383** | **0.399** | **-0.285** | **-0.839** | **0.551** | 0.011 | 0.107 | **0.438** |
| **25. RUE_E40InB** | **0.323** | 0.117 | **-0.691** | **-0.718** | 0.126 | 0.079 | **-0.99** | **-0.99** | **-0.812** | **0.819** | **0.99** | 0.012 | **0.286** | -0.016 | 0.045 | 0.108 |
| **26. RUE_InBA7** | **0.275** | 0.092 | -0.156 | **-0.323** | **-0.223** | **0.994** | **-0.388** | **-0.515** | **-0.516** | -0.124 | **0.380** | **0.261** | 0.054 | 0.144 | **0.245** | -0.102 |
| **27. RUE_GF** | **0.99** | **0.467** | **-0.990** | **-0.805** | **-0.814** | **-0.99** | 0.029 | **0.291** | -0.002 | **0.755** | **-0.608** | **-0.770** | **0.851** | **0.225** | **0.418** | **0.656** |
| **28. RUET** | **0.99** | **0.455** | **-0.977** | **-0.862** | **-0.559** | **-0.386** | **-0.606** | **-0.492** | **-0.600** | **0.645** | **0.222** | **-0.287** | **0.718** | -0.041 | 0.101 | **0.477** |
| **29. LIE40** | 0.014 | **0.422** | **0.207** | 0.12 | 0.024 | -0.058 | -0.006 | -0.036 | **-0.166** | -0.088 | -0.125 | -0.101 | **0.421** | **-0.425** | **-0.241** | **0.221** |
| **30. LIInB** | **-0.99** | **0.99** | **0.99** | **0.99** | **0.99** | **-0.99** | **0.99** | **0.99** | **0.99** | **-0.99** | **-0.99** | **-0.99** | **0.99** | **-0.99** | **-0.99** | **0.99** |
| **31. LIA7** | **-0.253** | **0.99** | **0.99** | **0.78** | **0.8** | -0.007 | **0.99** | **0.99** | **0.99** | -0.033 | **-0.99** | **-0.99** | **0.517** | **-0.902** | **-0.307** | **0.187** |

|  | **17** | **18** | **19** | **20** | **21** | **22** | **23** | **24** | **25** | **26** | **27** | **28** | **29** | **30** | **31** |
| --- | --- | --- | --- | --- | --- | --- | --- | --- | --- | --- | --- | --- | --- | --- | --- |
| **17. GWSP** | 1 |  |  |  |  |  |  |  |  |  |  |  |  |  |  |
| **18. SPKLSP** | **0.549** | 1 |  |  |  |  |  |  |  |  |  |  |  |  |  |
| **19. InfSPKLSP** | -0.064 | **0.644** | 1 |  |  |  |  |  |  |  |  |  |  |  |  |
| **20. Spike** | **0.487** | **0.714** | **0.202** | 1 |  |  |  |  |  |  |  |  |  |  |  |
| **21. BME40** | **-0.609** | **-0.695** | -0.071 | **-0.27** | 1 |  |  |  |  |  |  |  |  |  |  |
| **22. BMInB** | **-0.228** | **0.439** | **0.228** | 0.05 | **0.863** | 1 |  |  |  |  |  |  |  |  |  |
| **23. BMA7** | **-0.182** | -0.078 | **0.213** | 0.156 | **0.441** | **0.735** | 1 |  |  |  |  |  |  |  |  |
| **24. BMPM** | -0.016 | **0.373** | **0.327** | **0.399** | -0.141 | **0.582** | **0.552** | 1 |  |  |  |  |  |  |  |
| **25. RUE_E40InB** | -0.148 | **-0.579** | **-0.321** | **-0.337** | **0.555** | **0.786** | **0.99** | **0.380** | 1 |  |  |  |  |  |  |
| **26. RUE_InBA7** | -0.159 | **-0.314** | 0.023 | -0.077 | -0.146 | **-0.658** | **0.99** | 0.078 | **0.166** | 1 |  |  |  |  |  |
| **27. RUE_GF** | -0.045 | 0.032 | -0.066 | 0.072 | **-0.665** | **-0.400** | **-0.99** | **0.99** | **0.238** | **-0.99** | 1 |  |  |  |  |
| **28. RUET** | -0.146 | **-0.334** | **-0.177** | -0.034 | -0.025 | -0.014 | **0.825** | **0.99** | **0.99** | **0.639** | **0.99** | 1 |  |  |  |
| **29. LIE40** | **-0.201** | 0.013 | -0.015 | **0.258** | **0.99** | **0.828** | **0.332** | **0.22** | 0.136 | -0.024 | -0.038 | 0.098 | 1 |  |  |
| **30. LIInB** | **0.99** | **0.99** | **0.99** | **0.99** | **-0.99** | **0.99** | **0.99** | **0.99** | **-0.99** | **-0.99** | **0.99** | **-0.99** | **0.99** | 1 |  |
| **31. LIA7** | **-0.329** | **0.99** | **0.804** | **0.99** | **0.161** | **0.99** | **0.899** | **0.99** | **-0.99** | -0.05 | **0.99** | 0.052 | **0.99** | **0.99** | 1 |

**Table S4.** The distribution of the 35K Axiom Wheat Breeders Array loci on the Refseq1.0 Chinese Spring wheat physical map. Distribution of loci after anchoring loci associated sequences using BWA and inference from the consensus genetic map where sequences multi-mapped.

| A Genome | SNPs | B Genome | SNPs | D Genome | SNPs |
| --- | --- | --- | --- | --- | --- |
| 1A | 1,881 | 1B | 2,857 | 1D | 1,223 |
| 2A | 2,169 | 2B | 2,799 | 2D | 1,386 |
| 3A | 1,725 | 3B | 2,326 | 3D | 1,082 |
| 4A | 1,337 | 4B | 1,273 | 4D | 523 |
| 5A | 1,905 | 5B | 2,273 | 5D | 894 |
| 6A | 1,495 | 6B | 2,005 | 6D | 631 |
| 7A | 1,989 | 7B | 1,744 | 7D | 1,004 |
| A Total | 12,501 | B Total | 15,277 | D Total | 6,743 |

**Table S5.** The distribution of 35K Axiom array SNPs that were polymorphic in the HiBAP panel. Polymorphic loci distribution after filtering of MAF <5% and heterozygous calls.

| A Genome | SNPs | B Genome | SNPs | D Genome | SNPs |
| --- | --- | --- | --- | --- | --- |
| 1A | 438 | 1B | 910 | 1D | 316 |
| 2A | 623 | 2B | 784 | 2D | 299 |
| 3A | 451 | 3B | 631 | 3D | 166 |
| 4A | 350 | 4B | 273 | 4D | 57 |
| 5A | 559 | 5B | 755 | 5D | 108 |
| 6A | 486 | 6B | 628 | 6D | 112 |
| 7A | 591 | 7B | 570 | 7D | 160 |
| A Total | 3,498 | B Total | 4,551 | D Total | 1,218 |

**Table S6.** Linkage Disequilibrium/Decay statistics for the HiBAP panel. LD statistics based on pairwise comparisons of 9,424 SNP loci.

* where the panel specific critical LD is 0.301.

** *P* <0.01.

*** point at which LOESS regression falls below 0.301

|  | Pairwise Comparisons | Average R^2^ | Critical LD (%)* | Total LD (%) | In Significant LD (%)** | LD Decay (Mbp)*** |
| --- | --- | --- | --- | --- | --- | --- |
| All | 2,340,981 | 0.105 | 10.1 | 1.9 | 34.0 | 8.0 |
| A Genome | 782,725 | 0.96 | 9.3 | 1.2 | 31.7 | 7.0 |
| B Genome | 1,428,806 | 0.107 | 10.3 | 2.2 | 34.2 | 8.6 |
| D Genome | 129,449 | 0.132 | 13.0 | 3.5 | 34.3 | 12.4 |
| Chr1 | 540,717 | 0.134 | 13.2 | 4.9 | 34.0 |  |
| Chr2 | 433,597 | 0.124 | 12.7 | 1.4 | 40.4 |  |
| Chr3 | 252,037 | 0.09 | 9.4 | 0.7 | 31.7 |  |
| Chr4 | 84,950 | 0.124 | 12.2 | 2.4 | 36.4 |  |
| Chr5 | 425,415 | 0.081 | 6.9 | 1.1 | 26.4 |  |
| Chr6 | 305,334 | 0.092 | 8.5 | 0.5 | 33.7 |  |
| Chr7 | 298,931 | 0.81 | 7.0 | 0.6 | 32.1 |  |

**Table S7.** Summary of GWAS results. Common SNPs are indicated by the same colour.

| **Trait** | **SNP_ID** | **Chromosome** | **Position_(bp)** | **p-Value** | **Effect** |
| --- | --- | --- | --- | --- | --- |
| YLD | AX-95158872 | 5A | 37836752 | 0.00020 | 0.1 |
|  | AX-94518269 | 6A | 596085114 | 0.00076 | 0.08 |
|  | AX-94525122 | 7A | 68846954 | 0.00029 | 0.1 |
| Plm2 | AX-94428433 | 1A | 41710251 | 0.00075 | 0.08 |
|  | AX-94401499 | 2B | 711724837 | 0.00085 | 0.08 |
|  | AX-94648125 | 3B | 720991597 | 0.00049 | 0.08 |
|  | AX-94537094 | 5A | 35258899 | 0.00036 | 0.09 |
| Stm2_E40 | AX-94459558 | 2B | 63847339 | 0.00023 | 0.1 |
|  | AX-95201606 | 6B | 224865791 | 0.00013 | 0.12 |
| Stm2_InB | AX-94542766 | 1A | 590342675 | 0.00092 | 0.08 |
|  | AX-94659739 | 2D | 381335181 | 0.00090 | 0.08 |
|  | AX-94541408 | 3A | 655922676 | 0.00060 | 0.09 |
|  | AX-94526812 | 6B | 218026079 | 0.00001 | 0.16 |
| DTInB | AX-94987799 | 2B | 652615024 | 0.00013 | 0.12 |
|  | AX-95087924 | 3A | 686127775 | 0.00009 | 0.11 |
|  | AX-95209004 | 3D | 162182031 | 0.00046 | 0.09 |
|  | AX-94537943 | 5B | 591784516 | 0.00089 | 0.08 |
|  | AX-94443799 | 6B | 185673725 | 0.00017 | 0.1 |
| DTA | AX-95685212 | 2B | 141641152 | 0.00093 | 0.08 |
|  | AX-94987799 | 2B | 652615024 | 0.00015 | 0.11 |
|  | AX-95087924 | 3A | 686127775 | 0.00052 | 0.09 |
|  | AX-95209004 | 3D | 162182031 | 0.00049 | 0.09 |
|  | AX-94799130 | 3D | 338565962 | 0.00043 | 0.09 |
| RSGP | AX-94607341 | 1A | 3120046 | 0.00078 | 0.08 |
|  | AX-94432282 | 2B | 236882254 | 0.00006 | 0.12 |
|  | AX-94578116 | 2B | 546201896 | 0.00005 | 0.13 |
|  | AX-94803237 | 4D | 438144646 | 0.00076 | 0.09 |
| PGF | AX-95207530 | 3A | 538847476 | 0.00011 | 0.11 |
|  | AX-94455123 | 3A | 683193250 | 0.00039 | 0.09 |
|  | AX-94799130 | 3D | 338565962 | 0.00004 | 0.13 |
|  | AX-94431164 | 5B | 55184584 | 0.00093 | 0.08 |
| HI | AX-94728242 | 2B | 763862898 | 0.00072 | 0.08 |
|  | AX-94546552 | 6A | 557715847 | 0.00006 | 0.12 |
| TGW | AX-94659739 | 2D | 381335181 | 0.00099 | 0.08 |
|  | AX-95195224 | 6D | 91390591 | 0.00045 | 0.09 |
| GM2 | AX-94461881 | 2B | 691780758 | 0.00027 | 0.1 |
|  | AX-94385986 | 3B | 9936984 | 0.00095 | 0.08 |
|  | AX-95208994 | 5A | 505534395 | 0.00095 | 0.08 |
|  | AX-94777976 | 6D | 114233826 | 0.00053 | 0.09 |
|  | AX-95192986 | 7B | 328802260 | 0.00058 | 0.08 |
| SM2 | AX-94630858 | 1A | 92567883 | 0.00018 | 0.1 |
|  | AX-94873875 | 1A | 308813822 | 0.00055 | 0.08 |
|  | AX-94404955 | 1A | 402232673 | 0.00012 | 0.1 |
|  | AX-94846202 | 2B | 65370788 | 0.00084 | 0.08 |
|  | AX-94512327 | 3B | 21331548 | 0.00025 | 0.11 |
|  | AX-94797597 | 5B | 548428024 | 0.00096 | 0.07 |
|  | AX-95119680 | 6B | 223259475 | 0.00003 | 0.13 |
|  | AX-94409485 | 6B | 455081521 | 0.00059 | 0.09 |
|  | AX-94913939 | 7B | 647148119 | 0.00074 | 0.08 |
| GWSP | AX-94413932 | 1A | 462794136 | 0.00060 | 0.08 |
|  | AX-94540417 | 1B | 431456757 | 0.00034 | 0.09 |
|  | AX-94846202 | 2B | 65370788 | 0.00070 | 0.08 |
|  | AX-95119680 | 6B | 223259475 | 0.00002 | 0.14 |
| SPKLSP | AX-94651424 | 1A | 544723326 | 0.00044 | 0.09 |
|  | AX-95629283 | 2B | 593675095 | 0.00016 | 0.1 |
|  | AX-94567292 | 2B | 782122768 | 0.00013 | 0.1 |
|  | AX-95209004 | 3D | 162182031 | 0.00009 | 0.11 |
|  | AX-94552356 | 3D | 263354244 | 0.00044 | 0.1 |
|  | AX-95173991 | 4B | 649492569 | 0.00013 | 0.11 |
|  | AX-94512826 | 7A | 674277268 | 0.00035 | 0.09 |
| SpikeL | AX-95132498 | 5A | 619685320 | 0.00000 | 0.15 |
|  | AX-94539975 | 5B | 474973041 | 0.00004 | 0.13 |
|  | AX-94916259 | 7A | 129260071 | 0.00010 | 0.11 |
| BME40 | AX-94416856 | 1B | 22593095 | 0.00044 | 0.09 |
|  | AX-94407996 | 3B | 480568890 | 0.00027 | 0.1 |
| BMInB | AX-94884567 | 2A | 760619458 | 0.00049 | 0.09 |
|  | AX-94779538 | 4B | 665452374 | 0.00075 | 0.08 |
|  | AX-95080277 | 7A | 3907764 | 0.00028 | 0.1 |
| BM_PM | AX-95158872 | 5A | 37836752 | 0.00008 | 0.12 |
|  | AX-94522599 | 6A | 539866180 | 0.00030 | 0.09 |
|  | AX-94525122 | 7A | 68846954 | 0.00011 | 0.12 |
|  | AX-94958668 | 7A | 712423177 | 0.00038 | 0.09 |
|  | AX-94494622 | 7B | 709528334 | 0.00069 | 0.08 |
|  | AX-94880213 | 7D | 64270964 | 0.00058 | 0.09 |
| RUE_E40InB | AX-94861179 | 2A | 773562857 | 0.00001 | 0.14 |
|  | AX-95093243 | 2D | 645828094 | 0.00008 | 0.11 |
|  | AX-94796636 | 3B | 803624003 | 0.00004 | 0.12 |
|  | AX-94874193 | 6A | 520834218 | 0.00038 | 0.09 |
| RUE_GF | AX-94556635 | 1A | 33375656 | 0.00048 | 0.09 |
|  | AX-94425642 | 1D | 258388205 | 0.00075 | 0.08 |
|  | AX-94740180 | 2A | 676168828 | 0.00100 | 0.08 |
|  | AX-95158872 | 5A | 37836752 | 0.00004 | 0.13 |
|  | AX-95628663 | 6A | 52397115 | 0.00090 | 0.08 |
| RUET | AX-94781486 | 3D | 524870429 | 0.00056 | 0.09 |
|  | AX-95158872 | 5A | 37836752 | 0.00002 | 0.14 |
|  | AX-94440814 | 5A | 567522304 | 0.00060 | 0.08 |
|  | AX-94765421 | 6A | 592033900 | 0.00014 | 0.11 |
|  | AX-94525122 | 7A | 68846954 | 0.00057 | 0.1 |
| LIE40 | AX-94796636 | 3B | 803624003 | 0.00000 | 0.17 |
|  | AX-94491417 | 6D | 464403086 | 0.00055 | 0.08 |

**Table S8**. List of selected candidate genes found for the evaluated traits using GWA mapping. Results obtained from KnetMiner (http://knetminer.rothamsted.ac.uk/).

| **Trait** | **Chrom.** | **Input gene** | **Gene name** | **Evidence** |
| --- | --- | --- | --- | --- |
| BM_PM | 7A | TRIAE_CS42_7AL_TGACv1_556969_AA1774370 | SWEET5 | Klemens PAW et al. 2013 Plant Physiol |
|  |  | TraesCS7A01G533900 | Bidirectional sugar transporter SWEET | https://doi.org/10.1104/pp.113.224972 |
| BMInB | 7A | TRIAE_CS42_7AS_TGACv1_570274_AA1833190 | 1-SST | Xiang L et al 2013 Plant & Cell physiology |
|  |  | TraesCS7A01G010200 | Acid invertase 1 | https://doi.org/10.1093/pcp/pct075 |
| DTA/ DTInB | 2B | TRIAE_CS42_2BL_TGACv1_131212_AA0424460 | GTG2 | Transition from vegetative state, days to maturity |
|  |  | TraesCS2B01G458500 | Gpcr-type g protein 2 | Heo JB et al 2012 J. Biol.Chem 10.1074/jbc.M111.317412 |
| DTA | 2B | TRIAE_CS42_2BL_TGACv1_129782_AA0395690 | Calcium-binding EF hand protein | Pollen development |
|  |  | TraesCS2B01G458100 |  |  |
| DTA | 3D | TRIAE_CS42_3DS_TGACv1_272166_AA0915990 | PRL1 | Response to short day, transition from vegetative state |
|  |  | TraesCS3D01G181100 | WD40 repeat-like protein |  |
| DTA/PGF | 3D | TRIAE_CS42_3DL_TGACV1_249488_AA0849970 | Ribokinase | Associated with days to maturity, Expressed during anthesis |
|  |  | TraesCS3D01G242400 |  |  |
| GM2 | 2B | TRIAE_CS42_2BS_TGACv1_147406_AA0482770 | FOX3 | Grain size, average grain weight |
|  |  | TraesCS2B01G495000 | Retrotransposon |  |
| GM2 | 5A | TRIAE_CS42_5AL_TGACV1_374687_AA1206540 | AKR2B | Days to maturity, seed maturation |
|  |  | TraesCS5A01G296900 | Ankyrin repeat domain containing protein |  |
| GM2 | 5A | TRIAE_CS42_5AL_TGACv1_375268_AA1218910 | SNRNP35 | Seed maturation |
|  |  | TraesCS5A01G298000 | RNA-binding family protein |  |
| PGF | 3D | TRIAE_CS42_3AL_TGACv1_194606_AA0636680 | VCL1 | Seed maturation |
|  |  | TraesCS3D01G243600 | Vacuolar protein sorting-associated protein 16 |  |
| PGF | 5B | TRIAE_CS42_5BS_TGACv1_423287_AA1373440 | GER1 | Seed filling, Swatek et al 2011 J. Proteome Research, 10.1021/pr200263m |
|  |  | TraesCS5B01G048500 | Germin-like protein |  |
| RSGP | 1A | TRIAE_CS42_1AS_TGACv1_019727_AA0070660 | Receptor protein kinase | **positive regulation of growth**, response to red light, protein homooligomerization, pollen development, auxin homeostasis, HR, regulation of pollen tube growth, photomorphogenesis, polar cell growth, response to red or far red light |
|  |  | TraesCS1A01G005200 |  |  |
| RUET | 3D | TRIAE_CS42_3B_TGACv1_221740_AA0748940 | TFB1-1 | Response to UV |
|  |  | TraesCS3D01G412100 | BSD domain (BTF2-like transcription factors) |  |
| RUET | 3D | TRIAE_CS42_3DL_TGACv1_249354_AA0846360 | ALDH | Response to UV-B (chloroplast stroma) |
|  |  | TraesCS3D01G412700 | Aldehyde dehydrogenase |  |
| RUET/RUE_GF | 5A | TRIAE_CS42_5AS_TGACv1_393543_AA1273710 | CPT1 | response to light stimulus, response to blue light, chloroplast accumulation movement, blue light photoreceptor activity |
|  |  | TraesCS5A01G042600 | BTB/POZ domain-containing protein | [Haga K et al Plant Cell 2005 10.1105/tpc.104.028357](https://dx.doi.org/10.1105%2Ftpc.104.028357) |
| RUET/RUE_GF | 5A | TRIAE_CS42_5AS_TGACv1_393181_AA1269390 | GDI2 | Response to light stimulus, chloroplast stroma |
|  |  | TraesCS5A01G041100 | Guanosine nucleotide diphosphate dissociation inhibitor |  |
| RUET | 5A | TRIAE_CS42_5AS_TGACv1_392623_AA1262360 | TIF3A1 | response to light stimulus, response to low fluence blue |
|  |  | TraesCS5A01G042400 | Eukaryotic translation initiation factor 3 |  |
| RUE_E40InB | 2A | TRIAE_CS42_2AL_TGACV1_098345_AA0326010 | AAE3 | Chloroplast stroma |
|  |  | TraesCS2A01G581600 | Acetyl-coenzyme A synthetase |  |
| RUE_E40InB | 2D | TRIAE_CS42_2DL_TGACv1_160660_AA0552810 | GST3 | Response to UV-B, chloroplast stroma |
|  |  | TraesCS2D01G589600 | Glutathione S-transferase |  |
| RUE_E40InB | 2D | TRIAE_CS42_2DL_TGACv1_159456_AA0538510 | Ribulose bisphosphate carboxylase small chain, chloroplastic | Photosynthesis related |
|  |  | TraesCS2D01G587200 |  |  |
| RUE_E40InB | 6A | TRIAE_CS42_6AL_TGACv1_473240_AA1529550 | ALS | Chloroplast stroma |
|  |  | TraesCS6A01G288000 | Acetolactate synthase | Riethmuller-Haage I et al 2006 Photosyn Res. https://link.springer.com/article/10.1007%2Fs11120-006-9062-z |
| RUE_E40InB | 6A | TRIAE_CS42_2DL_TGACv1_157935_AA0502550 | TET11 | Photosynthesis |
|  |  | TraesCS6A01G289800 | Tetraspanin | [Wang J et al 2017 Nuc Acid Res 10.1093/nar/gkx869](https://dx.doi.org/10.1093%2Fnar%2Fgkx869) |
| RUE_E40InB | 6A | TRIAE_CS42_6AL_TGACv1_474358_AA1535080 | BZIP43 | Response to far red light, response to red light |
|  |  | TraesCS6A01G288900 | BZIP transcription factor |  |
| RUE_GF | 1A | TRIAE_CS42_1AS_TGACv1_019485_AA0067220 | WEB family protein, chloroplastic | Required for the chloroplast avoidance response under high intensity blue light (EBI) |
|  |  | TraesCS1A01G051300 |  |  |
| RUE_GF | 1D | TRIAE_CS42_1BL_TGACv1_031864_AA0121960 | OXA1 | [Alb3/Oxa1/YidC family involved exclusively in insertion and/or assembly of subunits of chlorophyll-containing photosynthetic complexes. Benz M et al, 2009, Mol Plant, https://doi.org/10.1093/mp/ssp095](https://doi.org/10.1093/mp/ssp095) |
|  |  | TraesCS1D01G186800 | Mitochondrial inner membrane protein |  |
| RUE_GF | 1D | TRIAE_CS42_1DL_TGACv1_063356_AA0226600 | OXA1L | [Alb3/Oxa1/YidC family involved exclusively in insertion and/or assembly of subunits of chlorophyll-containing photosynthetic complexes. Benz M et al, 2009, Mol Plant, https://doi.org/10.1093/mp/ssp095](https://doi.org/10.1093/mp/ssp095) |
|  |  | TraesCS1D01G186700 | Membrane protein insertase YidC |  |
| RUE_GF | 6A | TRIAE_CS42_6AS_TGACv1_485890_AA1553720 | Response to low sulfur protein | Water use efficiency, chloroplast stroma, response to UV, photosynthetic electron transport, response to light stimulus, response to ionizing radiation, response to blue light, response to far red light, reductive pentose-phosphate, photosynthesis, response to UV-B, response to red light, photorespiration |
|  |  | TraesCS6A01G085000 |  |  |
| RUE_GF | 6A | TRIAE_CS42_6AS_TGACv1_486346_AA1560070 | Early light-inducible protein (ELIP) | photosynthesis, light harvest, photosynthesis, photosystem II |
|  |  | TraesCS6A01G084200 |  |  |
| RUE_GF | 6A | TRIAE_CS42_6AS_TGACv1_488350_AA1575250 | PDX2 | chloroplast stroma, endomembrane system, response to UV-B |
|  |  | TraesCS6A01G084600 | Pyridoxal 5'-phosphate synthase subunit PdxT |  |
| RUE_GF | 6A | TRIAE_CS42_6AS_TGACv1_487658_AA1572190 | PIP2-3 | Endomembrane system, linked to total biomass yield |
|  |  | TraesCS6A01G082900 | Aquaporin |  |
| SM2 | 1A | TRIAE_CS42_1AS_TGACv1_019004_AA0057730 | TGA5 | Culm number |
|  |  | TraesCS1A01G096300 | transcription factor-like protein (seed dormancy control) |  |
| SM2 | 1A | TRIAE_CS42_1DL_TGACv1_061238_AA0189630 | Ras family protein | Culm number |
|  |  | TraesCS1A01G172300 |  |  |
| SM2 | 7B | TRIAE_CS42_7DL_TGACv1_604439_AA1998160 | RAN1 | Tiller number |
|  |  | TraesCS7B01G382300 | Copper-transporting ATPase |  |
| SPKLSP | 4B | TRIAE_CS42_4BL_TGACv1_322260_AA1070160 | FAD-binding Berberine family protein | spikelet fertility, average grain weight, heat tolerance |
|  |  | TraesCS4B01G358600 |  |  |
| TGW | 2D | TRIAE_CS42_2DL_TGACv1_159390_AA0537610 | WAT1-related protein | Seed weight, see maturation |
|  |  | TraesCS2D01G298200 |  |  |
| TGW | 6D | TRIAE_CS42_6DS_TGACv1_543659_AA1742730 | Peroxidase | Negative correlation between peroxidase activity and grain weight: Singhall NC et al 1979, Theor. Appl. Genetics. https://link.springer.com/article/10.1007%2FBF00285196 |
|  |  | TraesCS6D01G127100 |  |  |
| TGW | 6D | TRIAE_CS42_6DS_TGACv1_542633_AA1725840 | ARF6 | Pollen development, seed weight, brown rice shape |
|  |  | TraesCS6D01G127600 | Auxin response factor |  |

**Fig. S1**. Histograms of the distribution of the phenotypic values of plant height, anthesis date (DTA) and days to maturity (DTM) from the combined analysis from two years’ evaluation (Y16 and Y17) in NW Mexico.

**Figure S2.** Boxplots of the best linear estimated predictions (BLUEs) of the main traits measured in HiBAP during two years of evaluation (Y16&Y17). BM_PM biomass at physiological maturity, HI harvest index, TGW thousand grain weight, GM2 grains per square meter, DTA days to anthesis, DTM days to maturity. Box-whisker plots report the distribution of the data. The lower and upper part of the rectangles (box) give the estimated 25th and 75th percentile, the line in the middle indicates the median and the dots the outliers. The middle dotted line is the adjusted mean across lines.


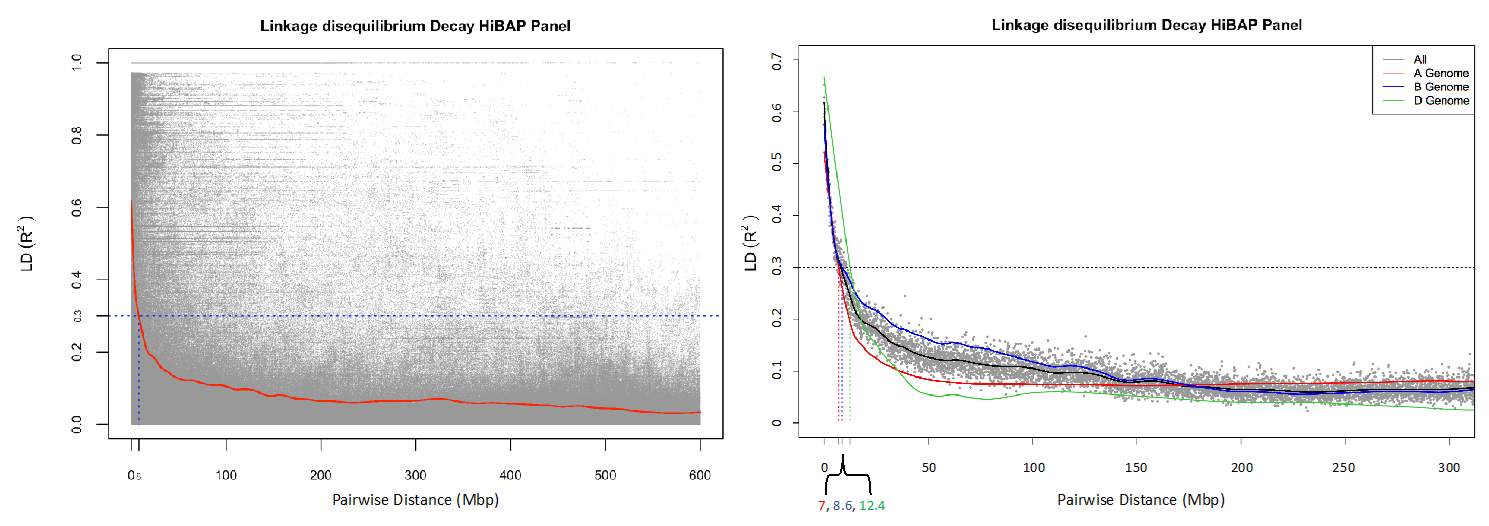


**Figure S3.** Linkage Disequilibrium Decay depicted as a scatter plot of pairwise SNP LD (R^2^) and pairwise physical distance across the hexaploid wheat genome. **Left**: Pairwise R^2^ values for >2.3 million intra-chromosomal comparisons with a maximum distance of 600Mbp. The red line represents a local polynomial regression LOESS curve indicator of LD decay. The blue dashed line represents the 95^th^ percentile of R^2^ distribution of unlinked pairwise comparisons, taken as the population specific critical LD in this study (0.301). **Right**: A scatter plot of pairwise R^2^ values averaged across 50 Kbp bins. The red, blue, green and black LOESS curves depict LD decay over physical distance for the A, B, D and whole genome respectively. The dashed line represents the population specific critical LD value.

**Figure S4.** GWAS results using 21,708 SNPs markers in HiBAP for yield traits based on BLUEs means obtained from the combined analysis from Y16 and Y17. The dotted horizontal line indicates threshold of significance. The traits appear in alphabetical order.


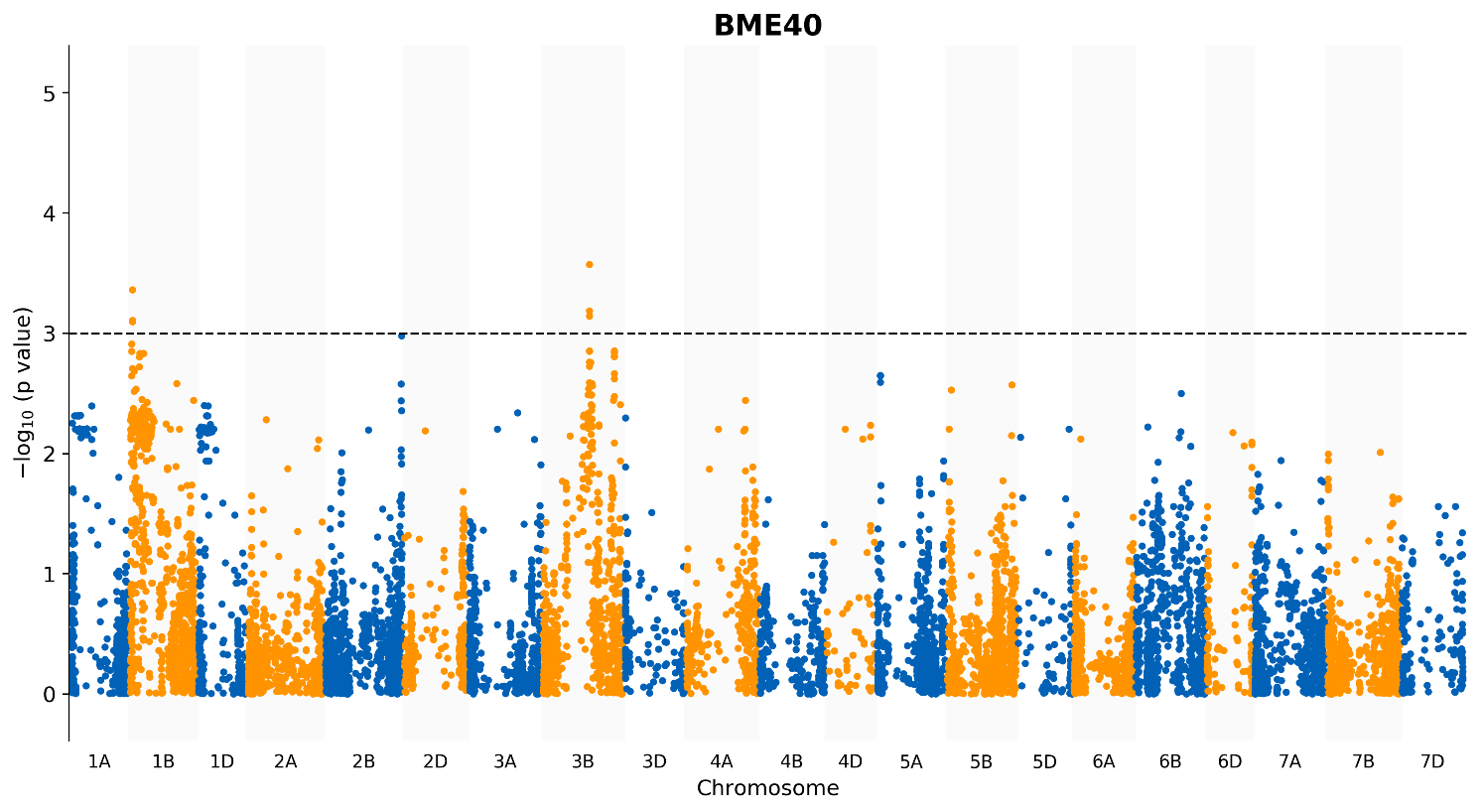


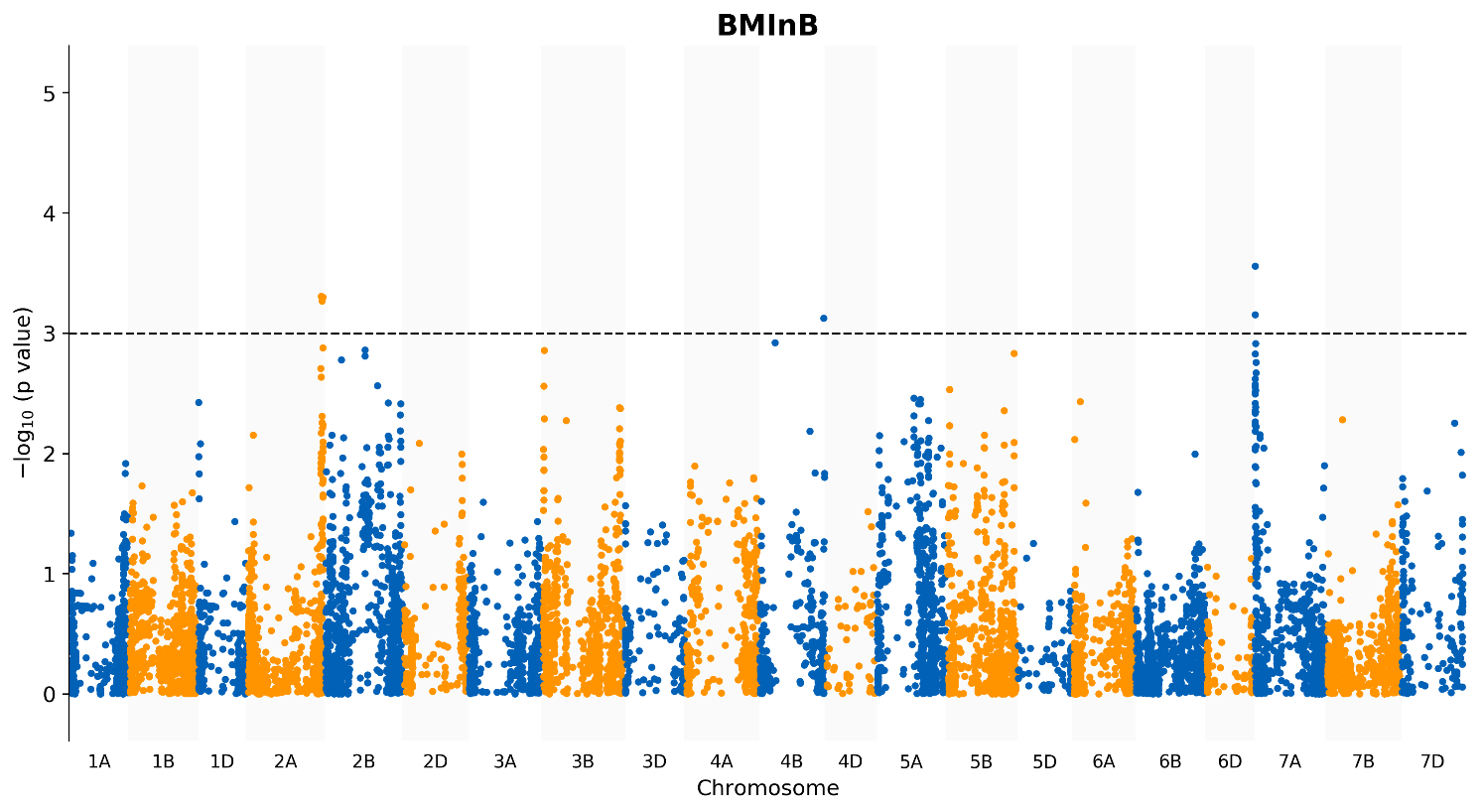


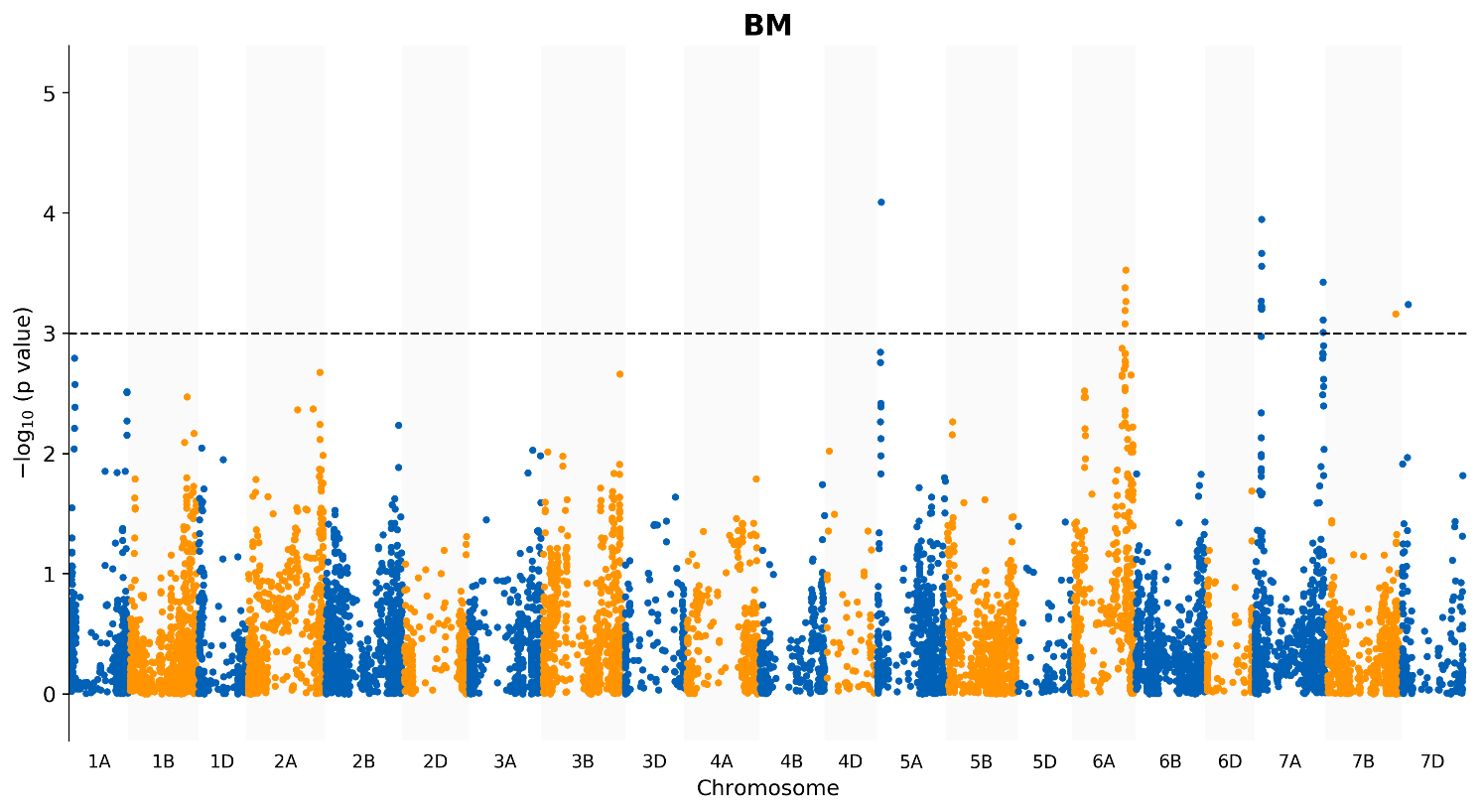


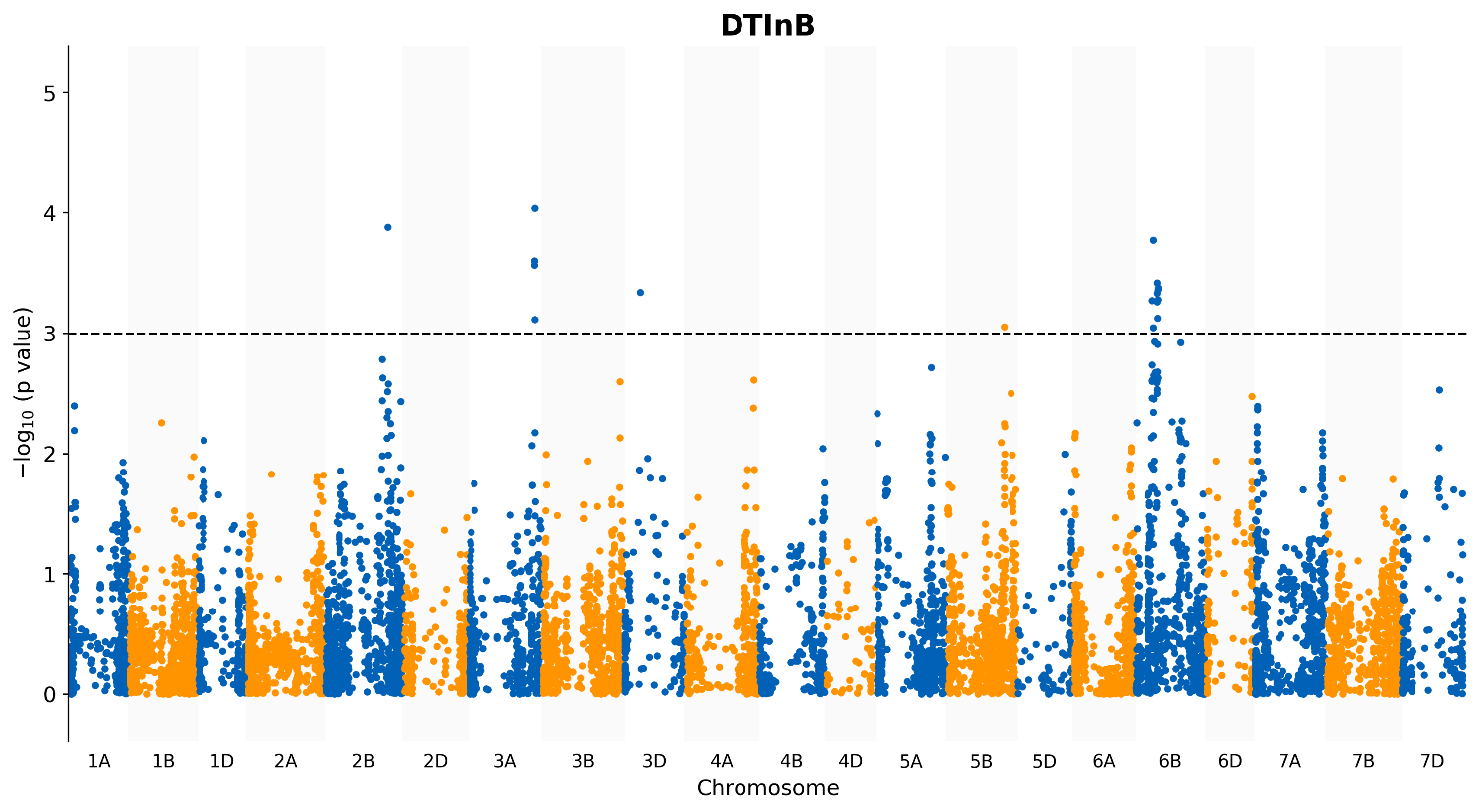


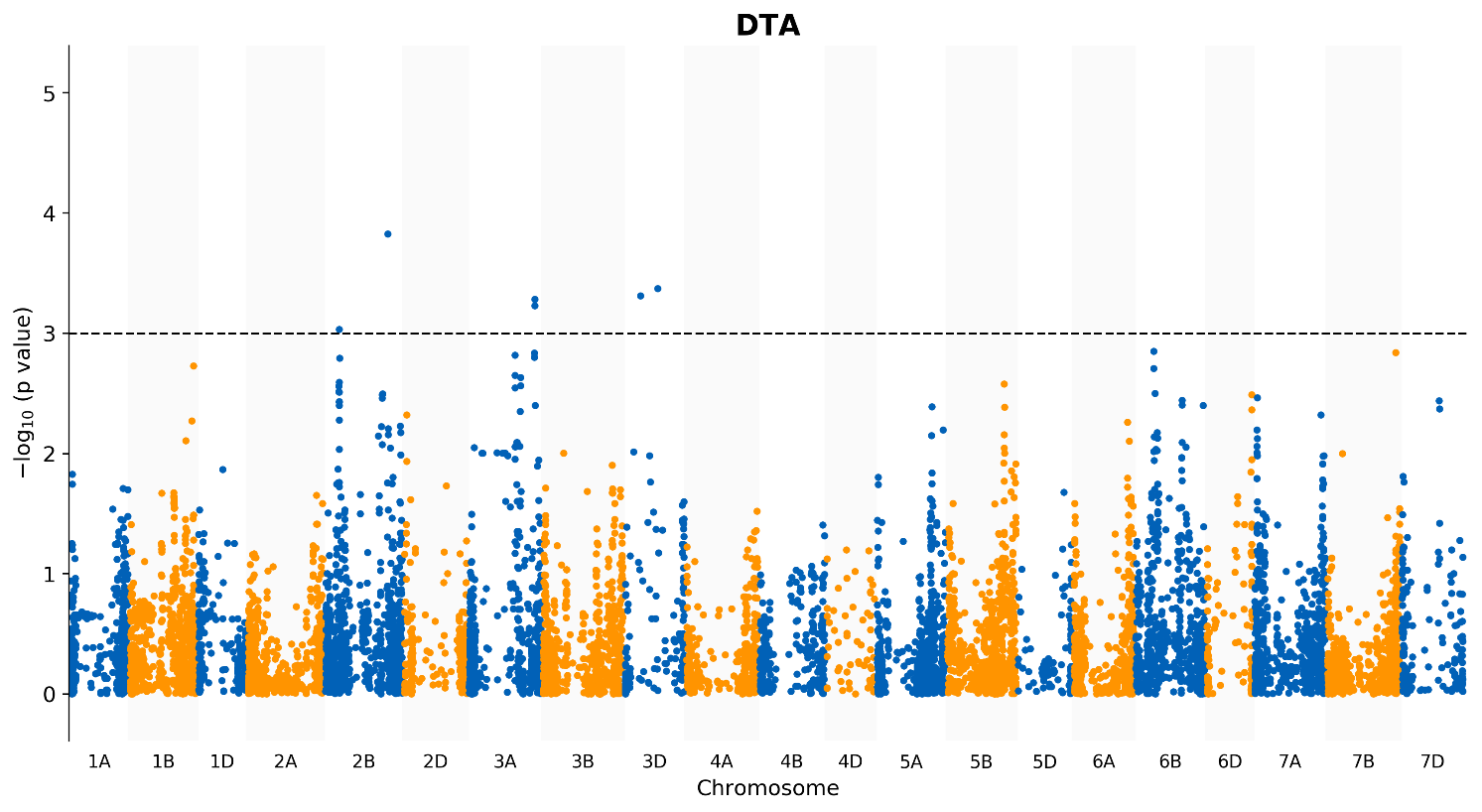


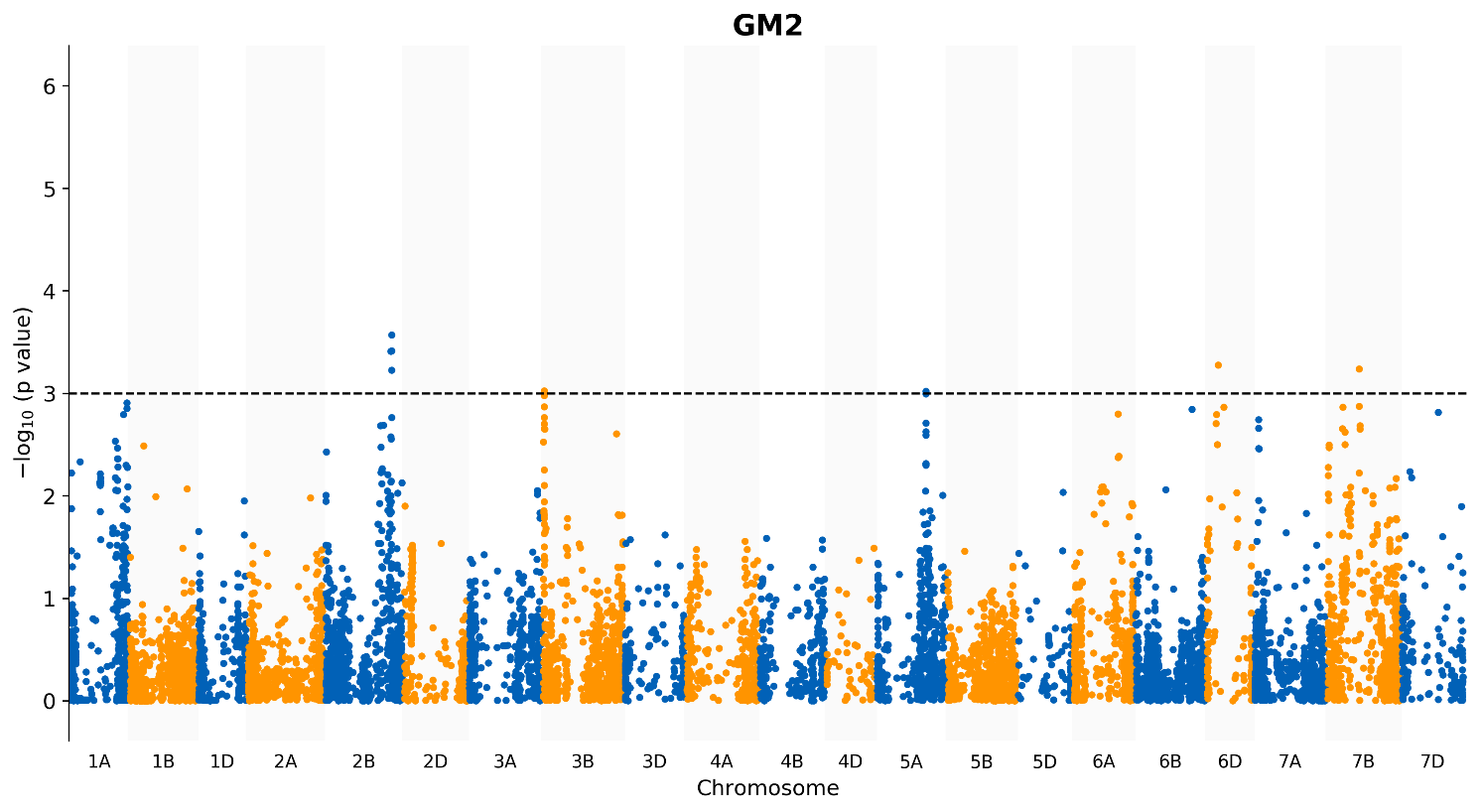


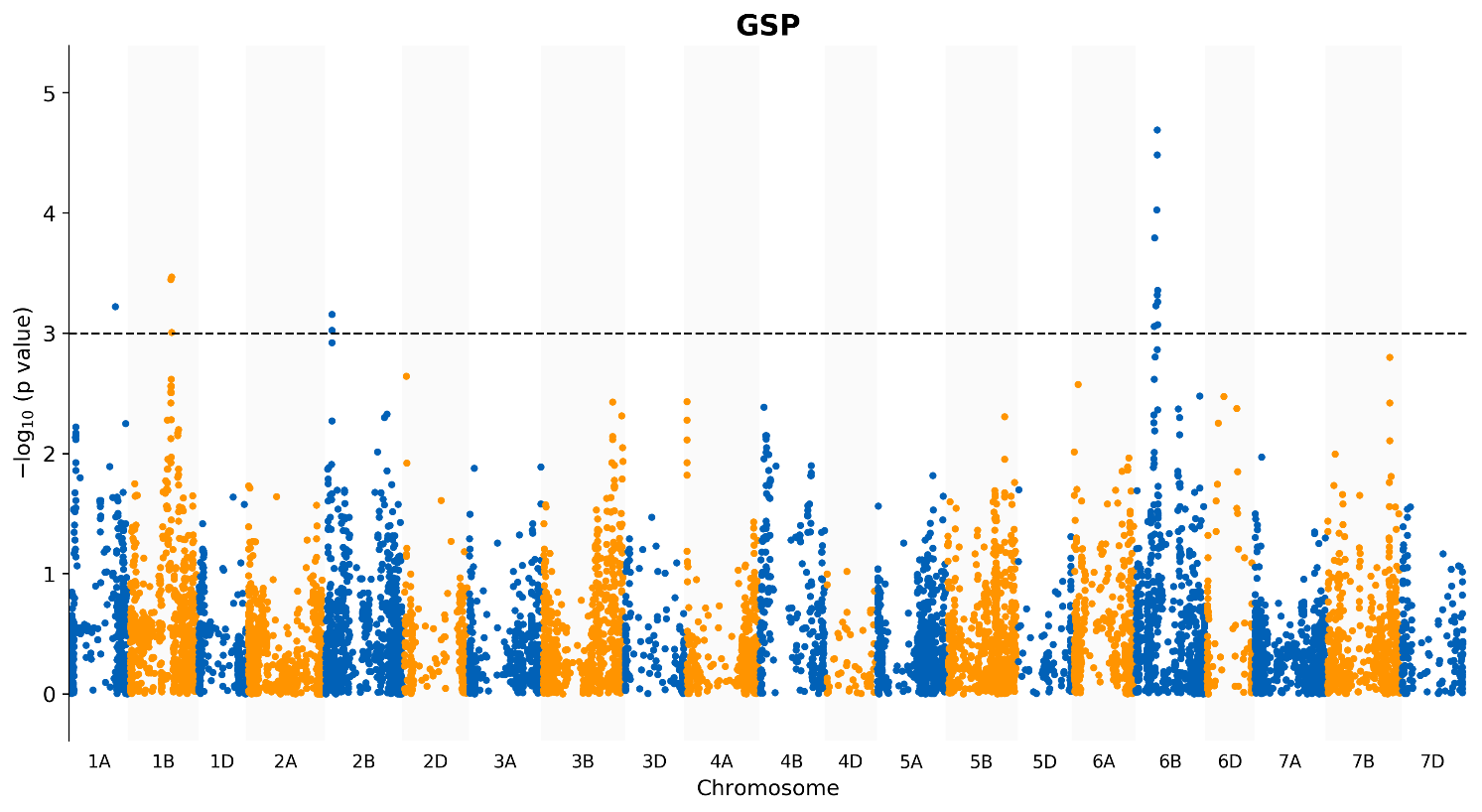


**GWSP**


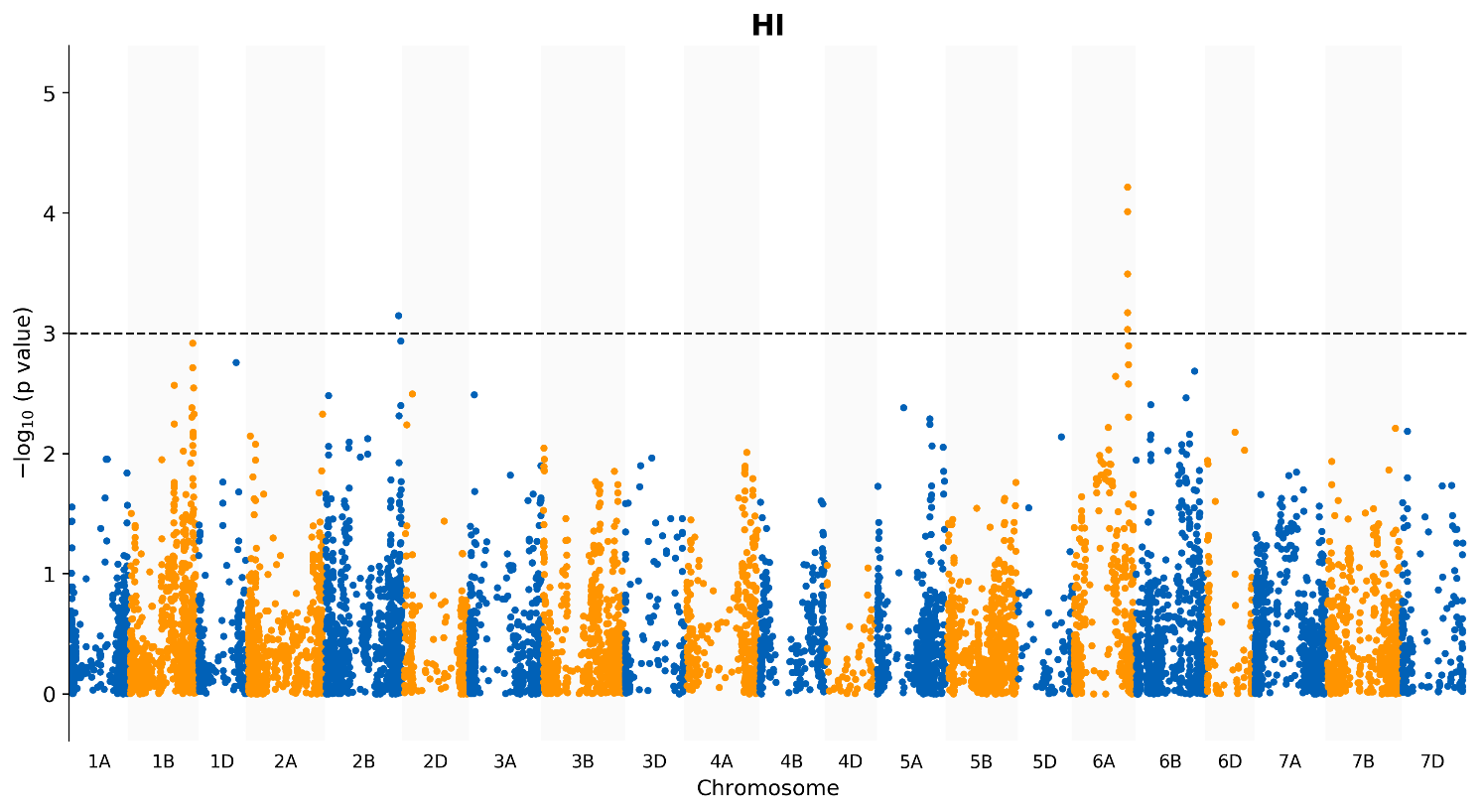


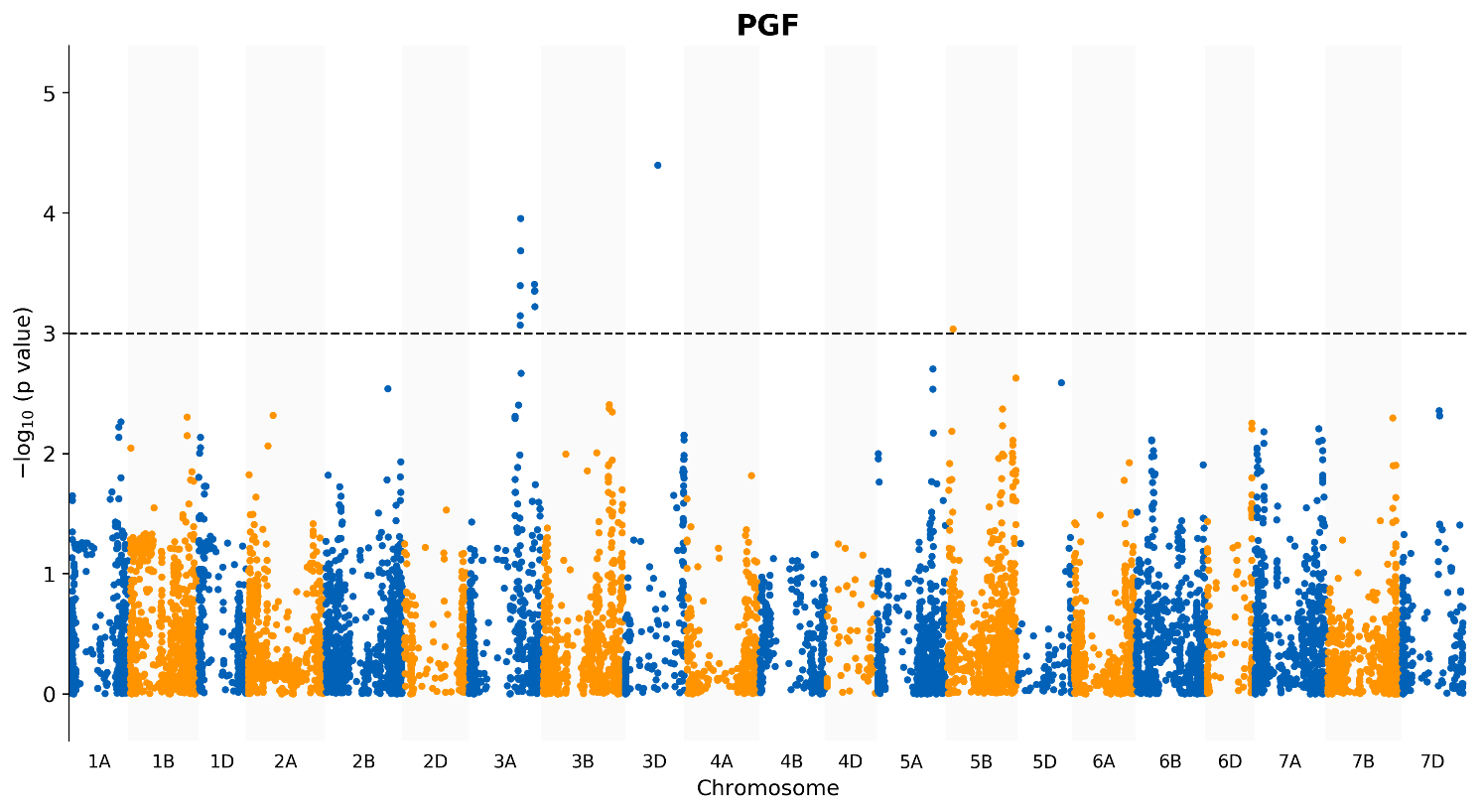


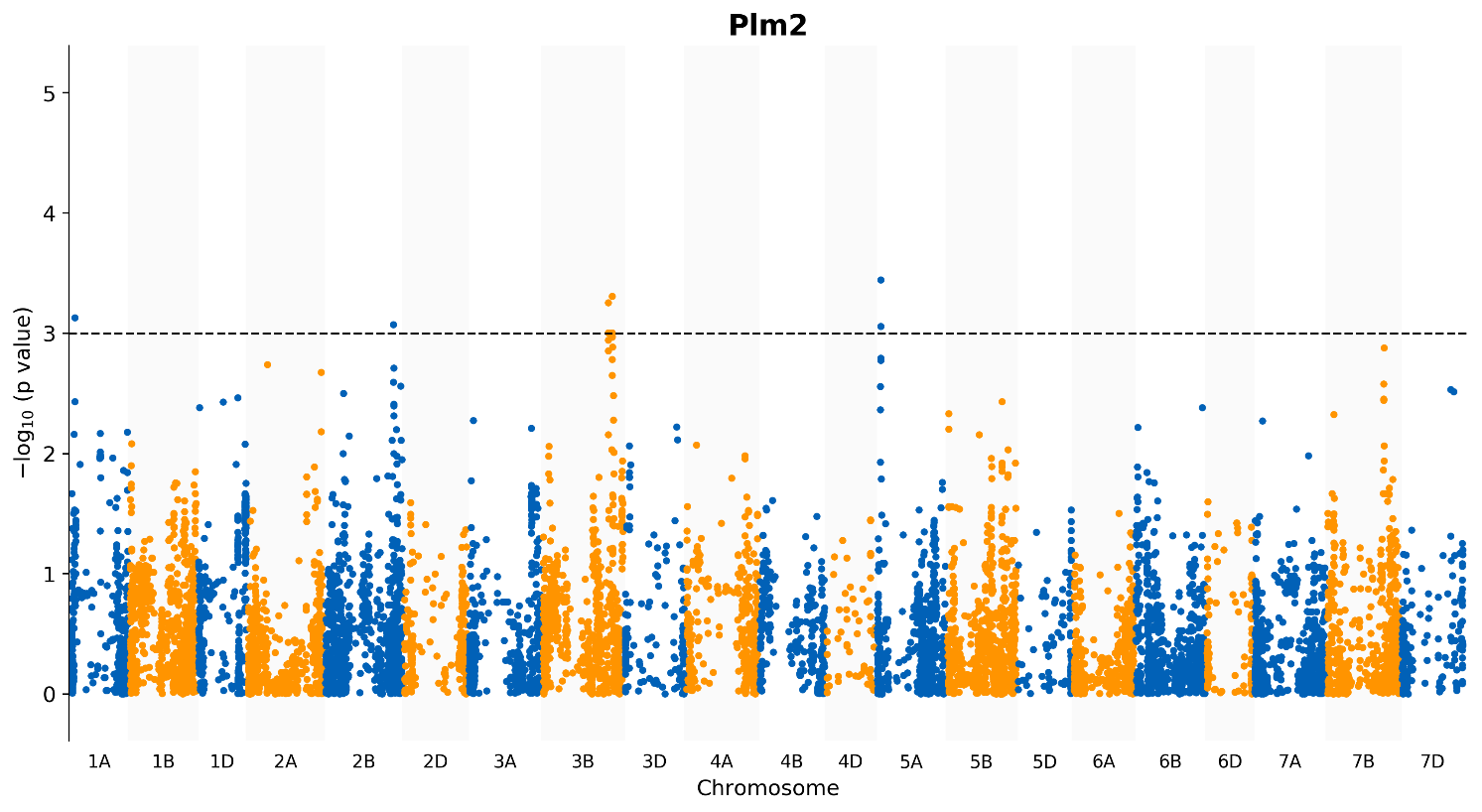


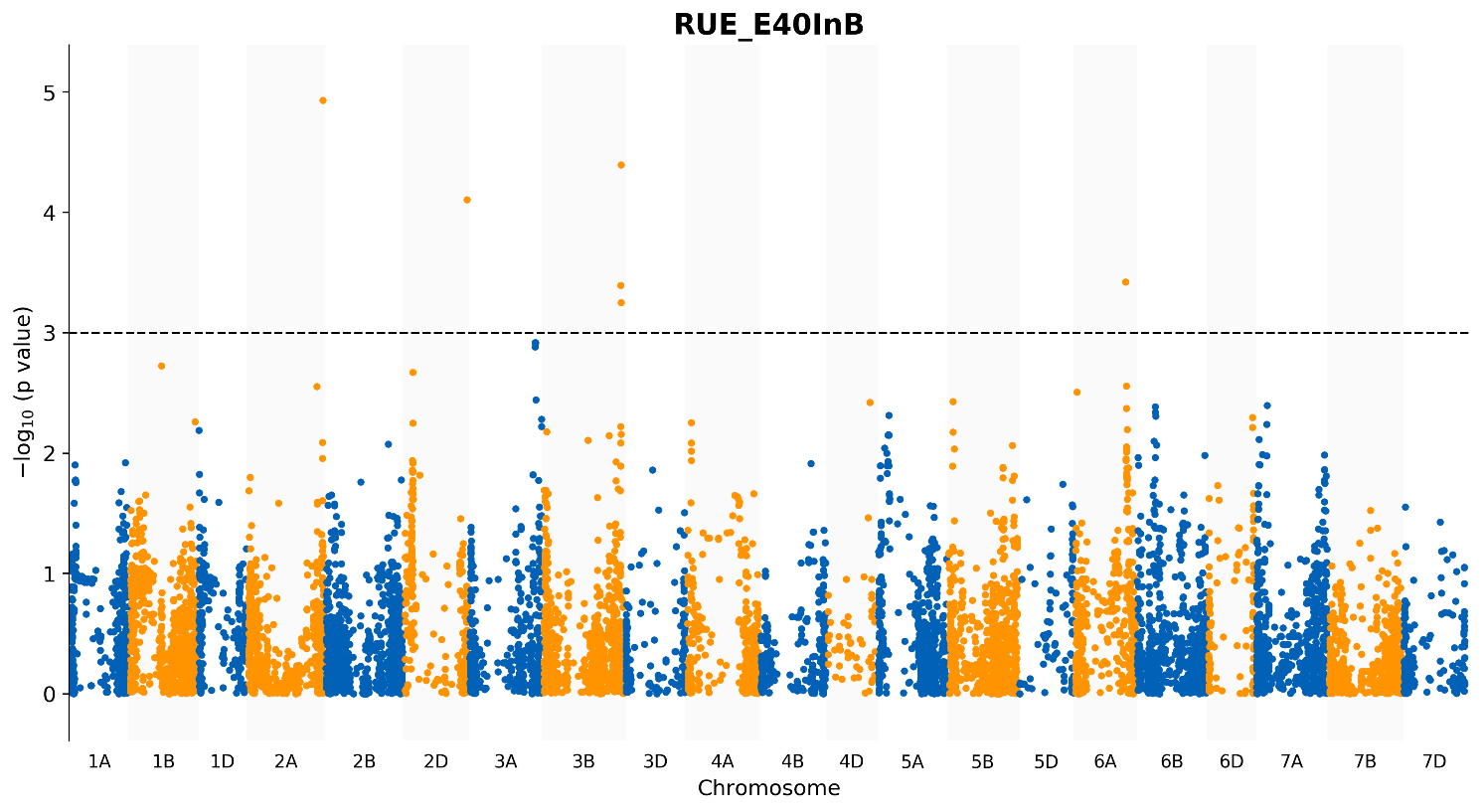


**RUE_GF**


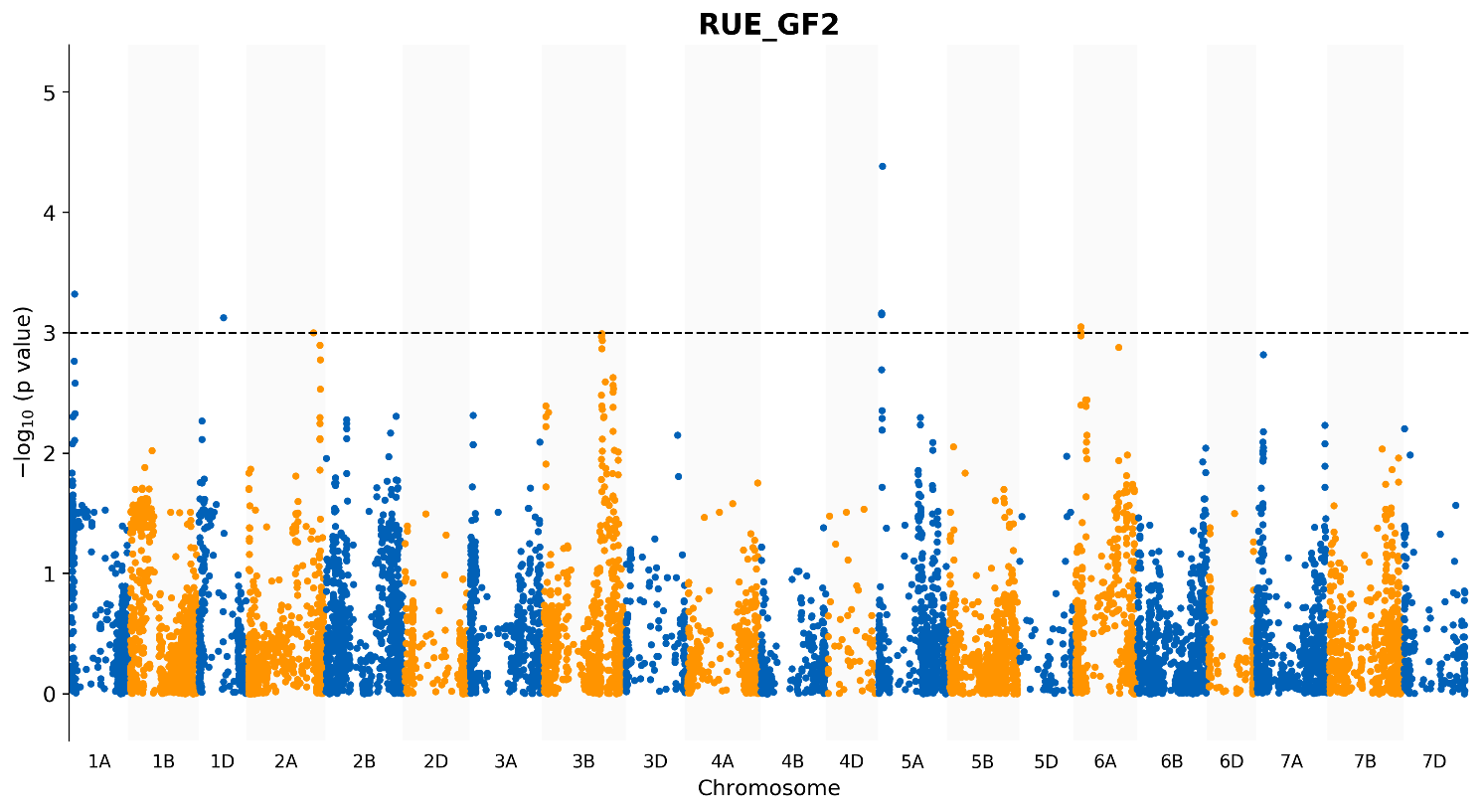


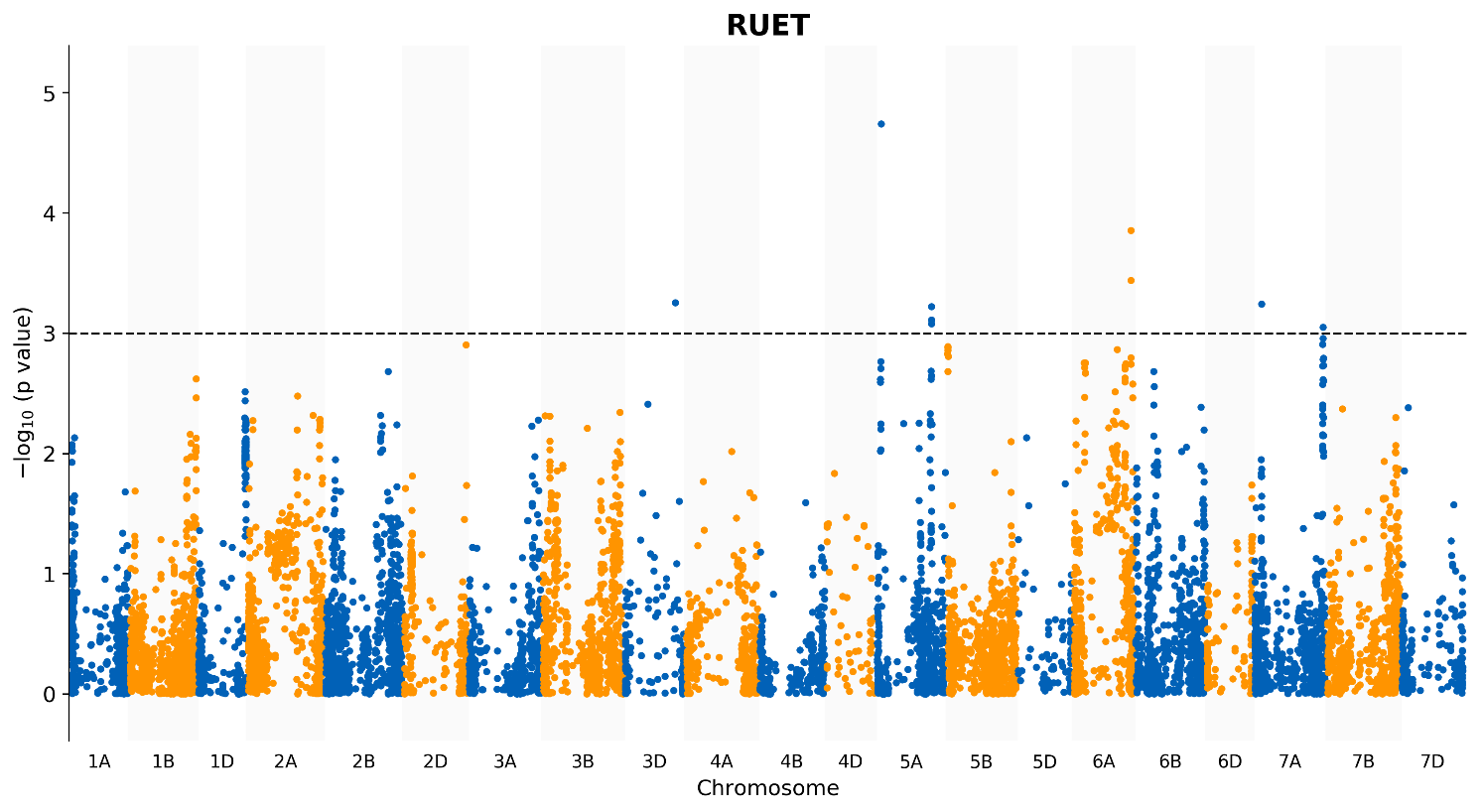


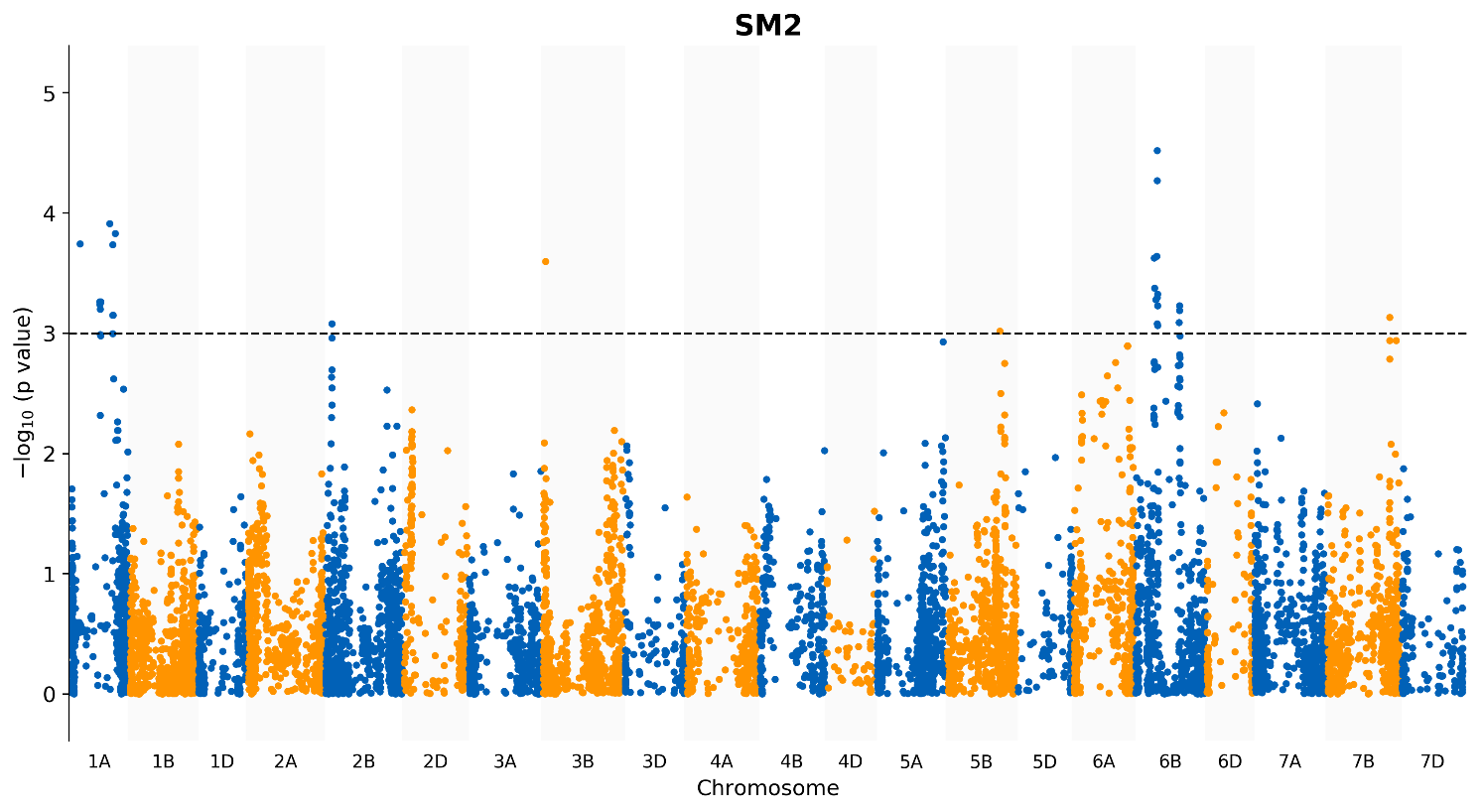


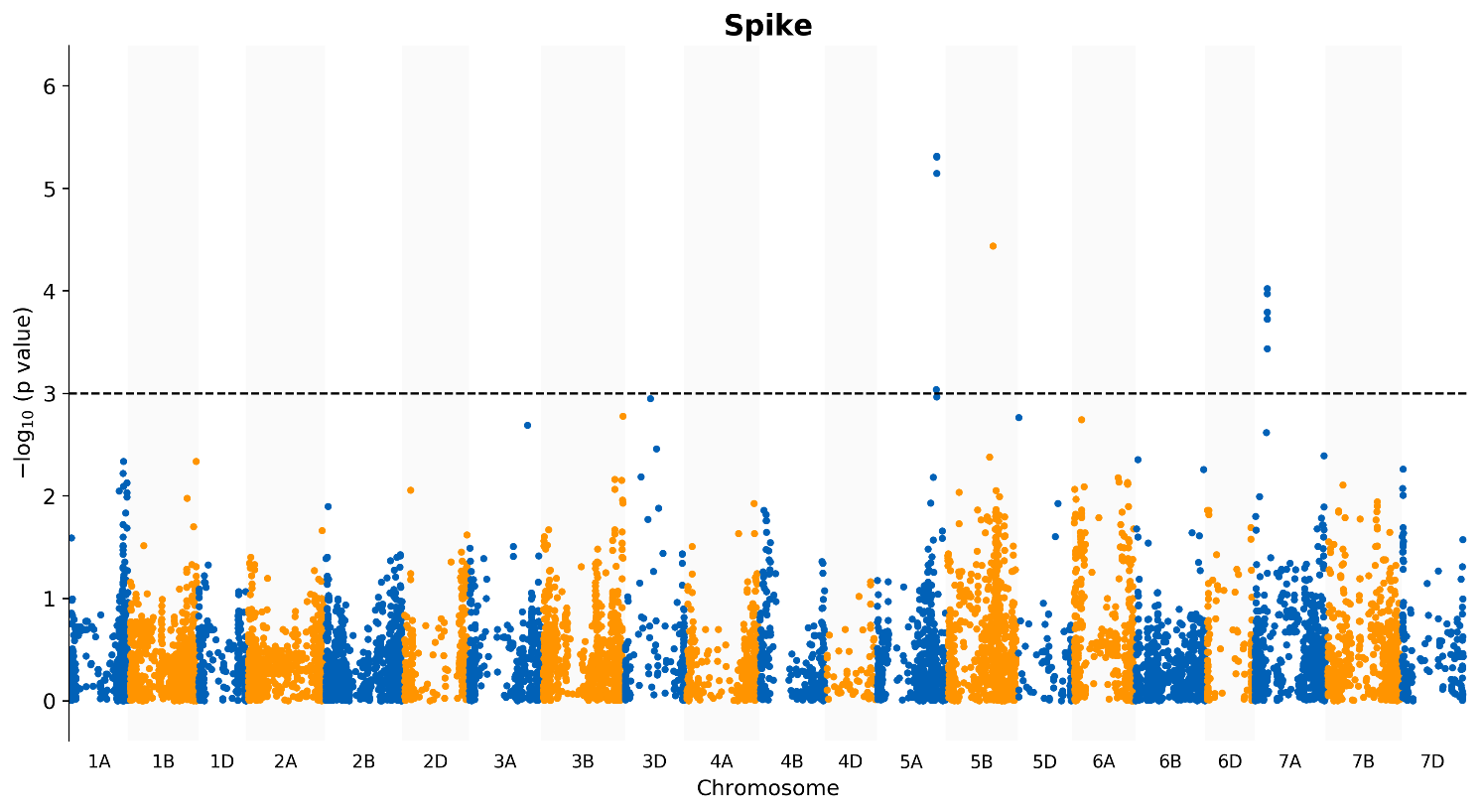


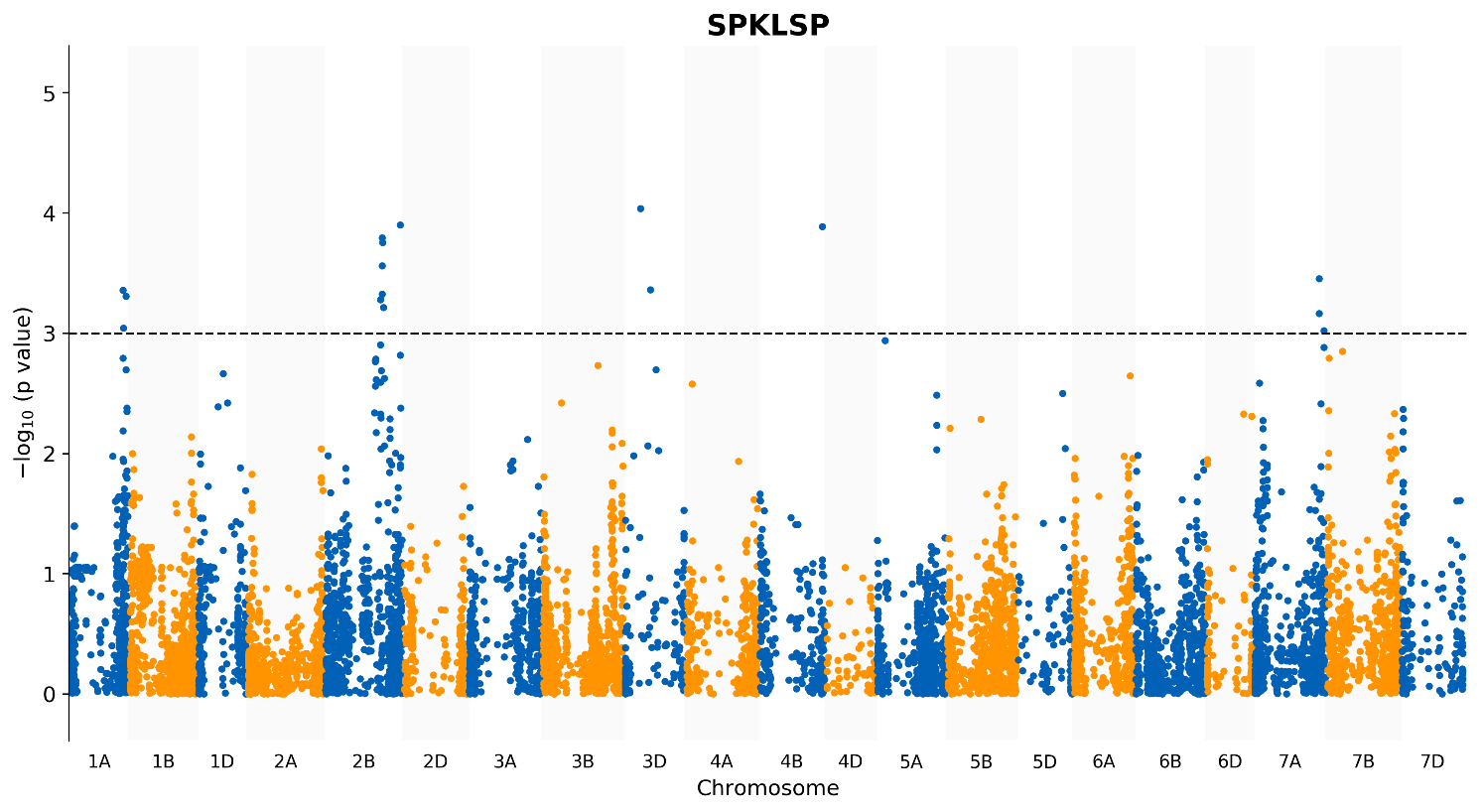


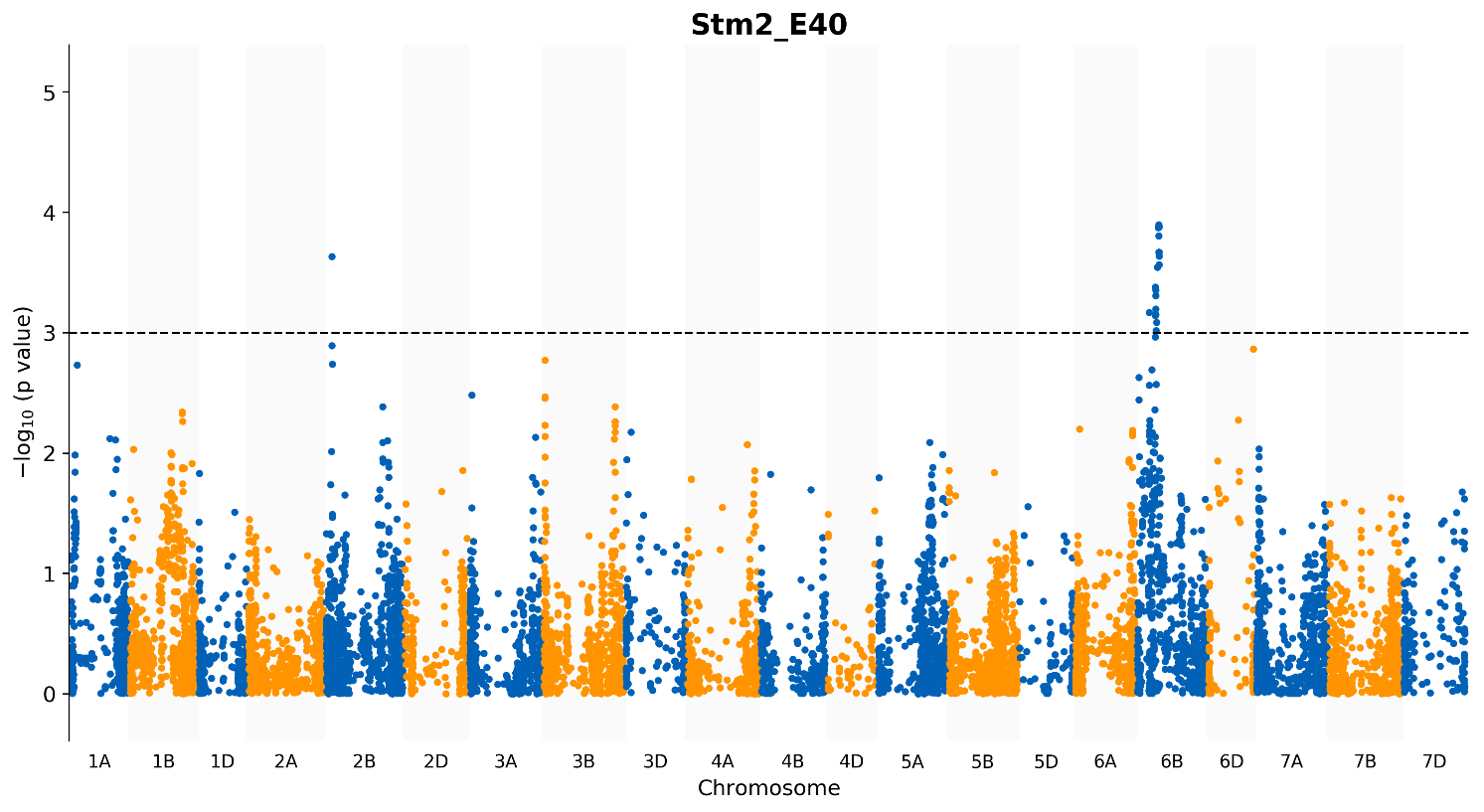


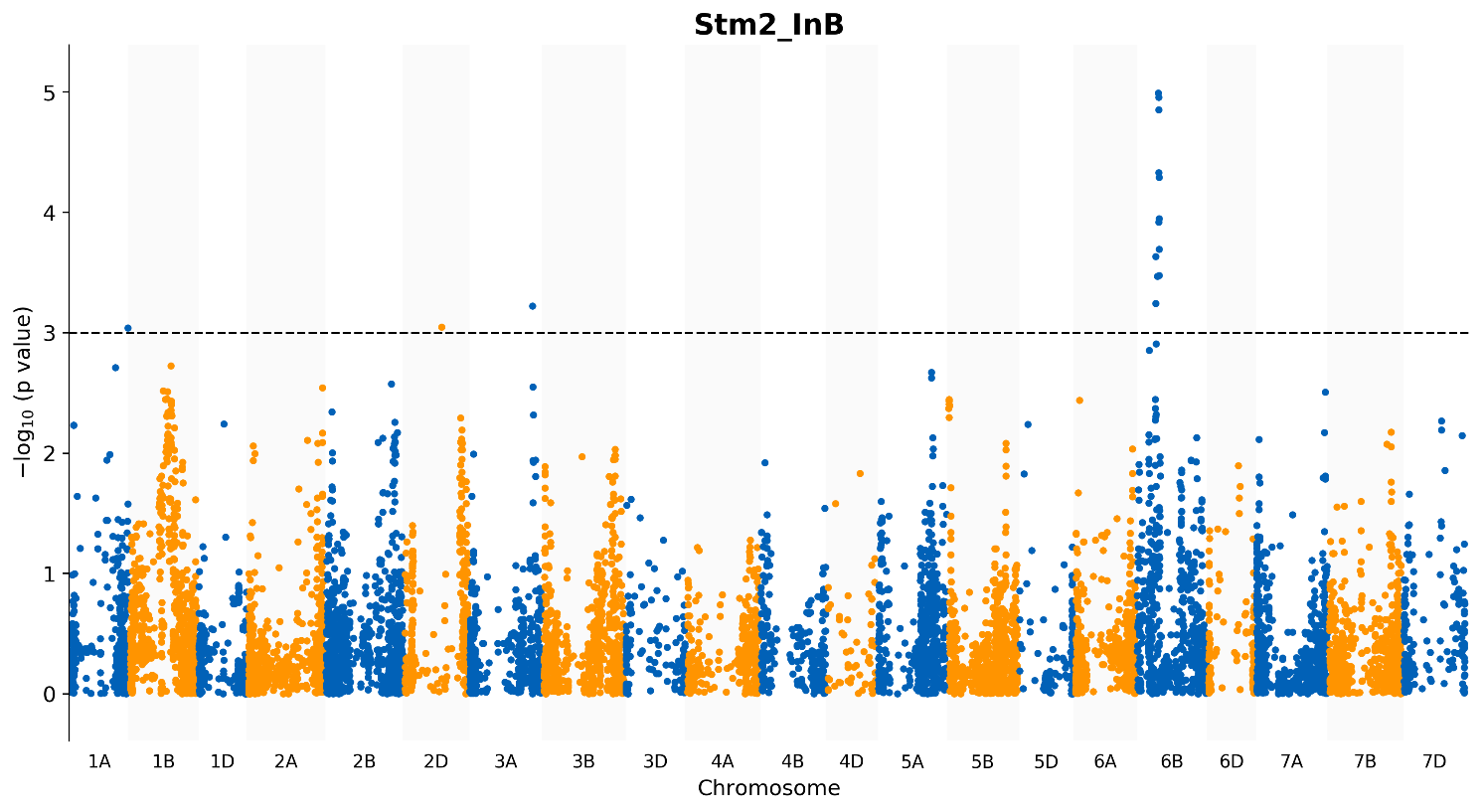


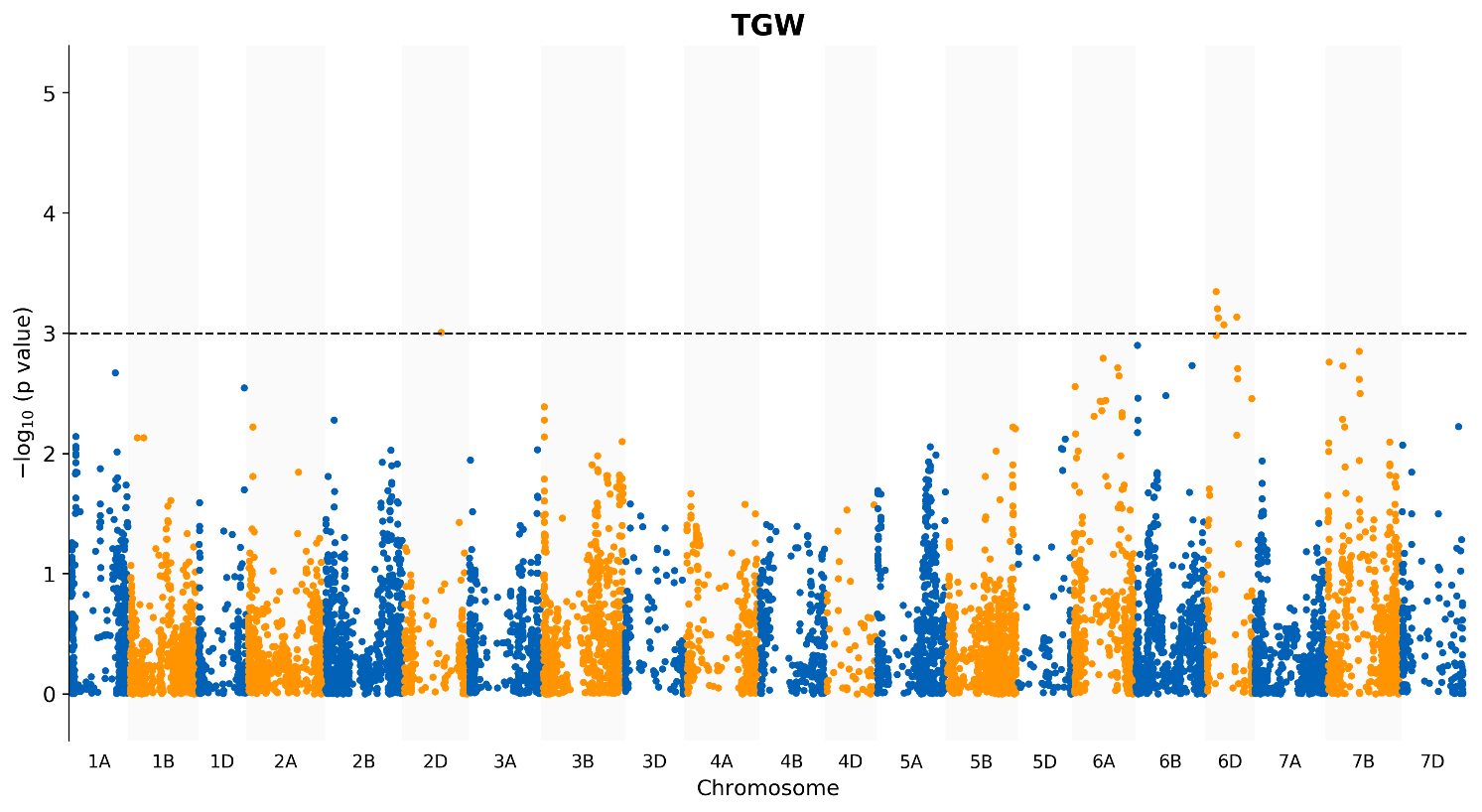


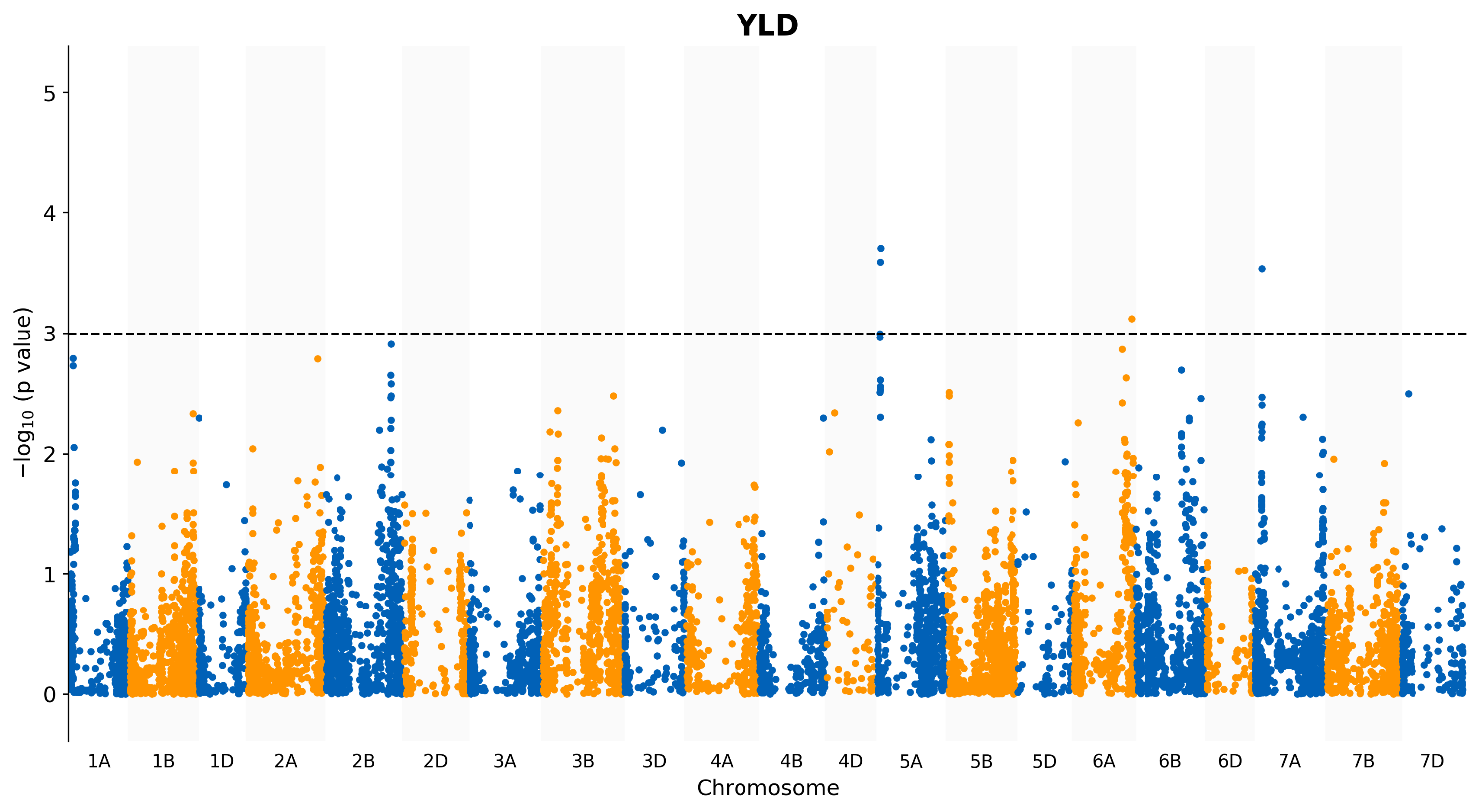


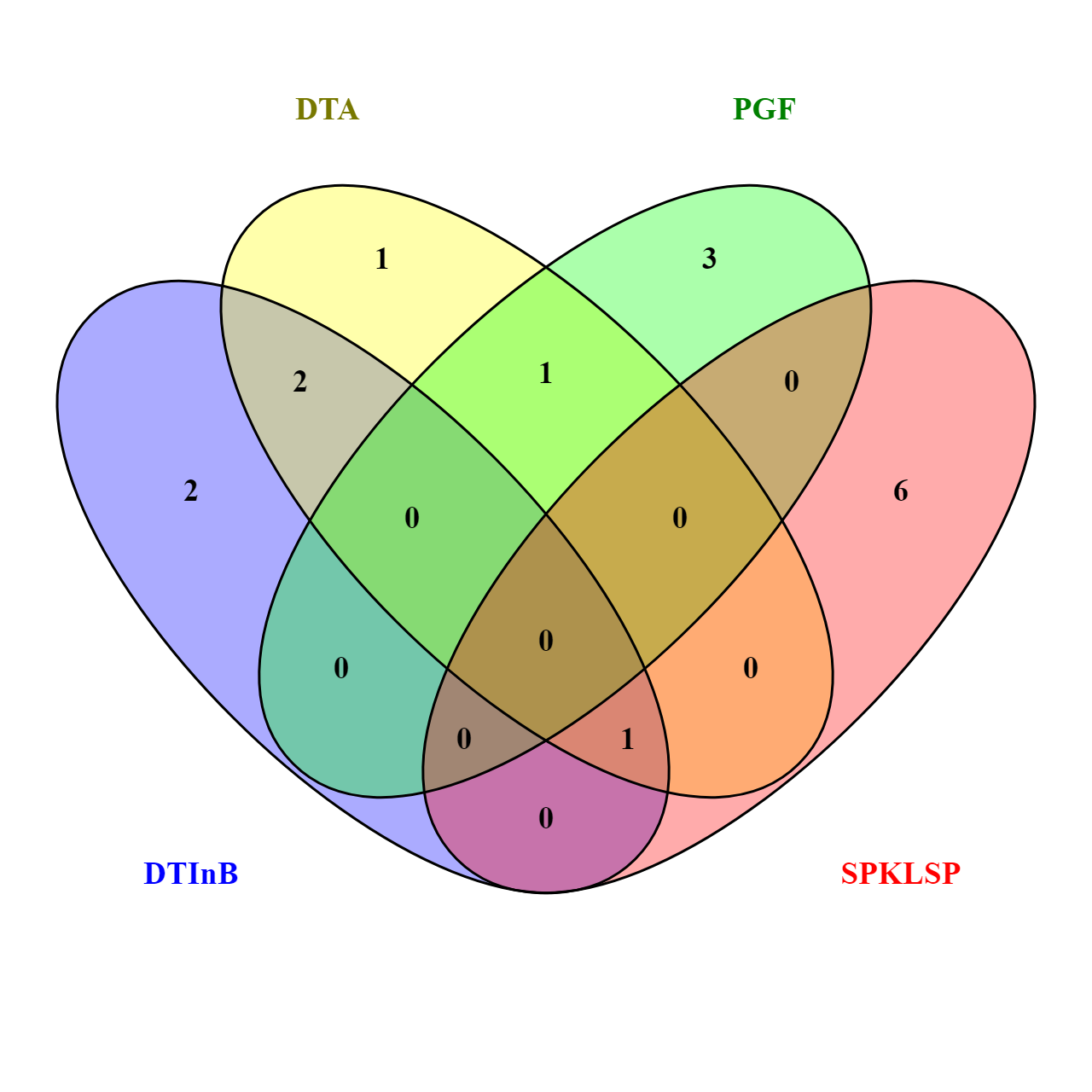

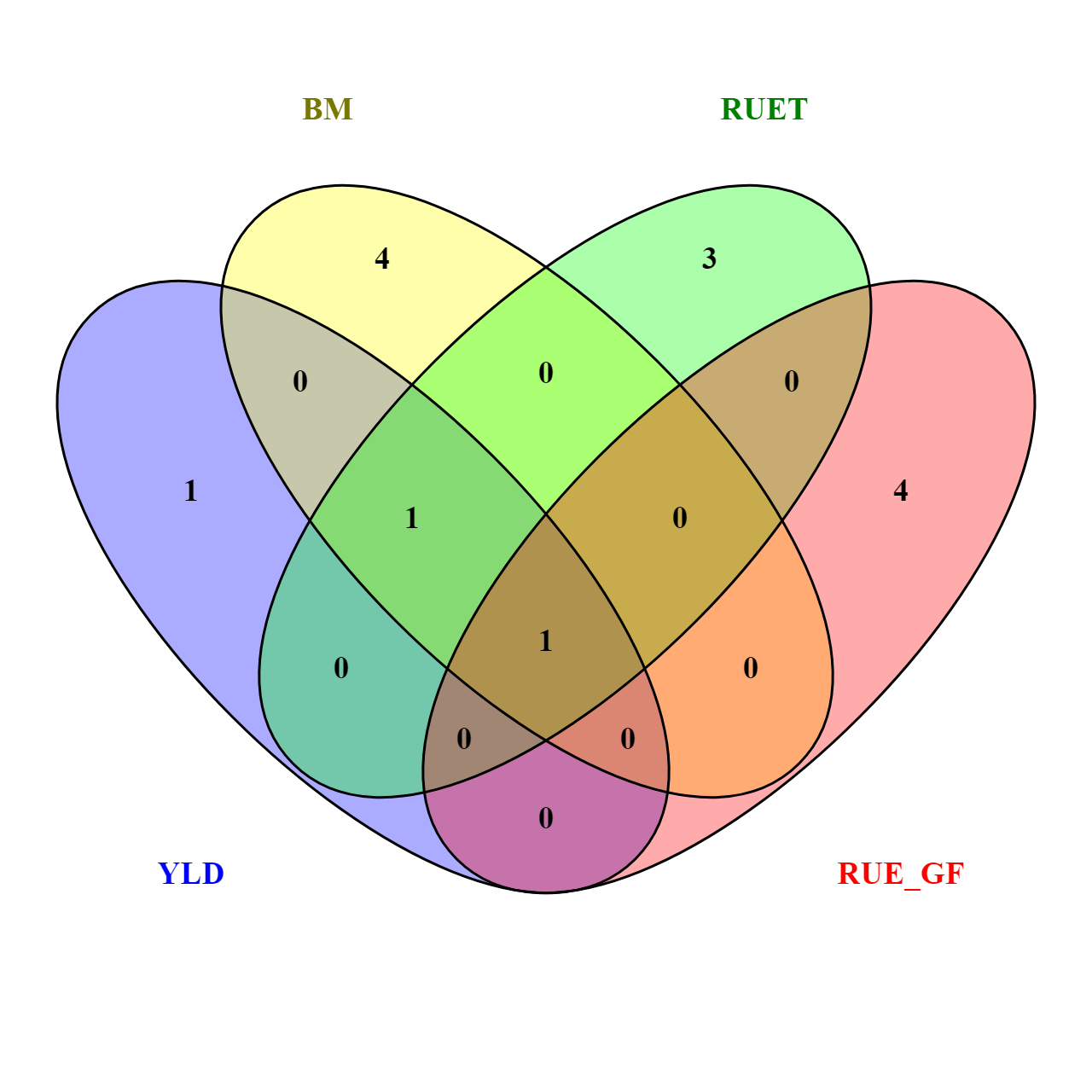

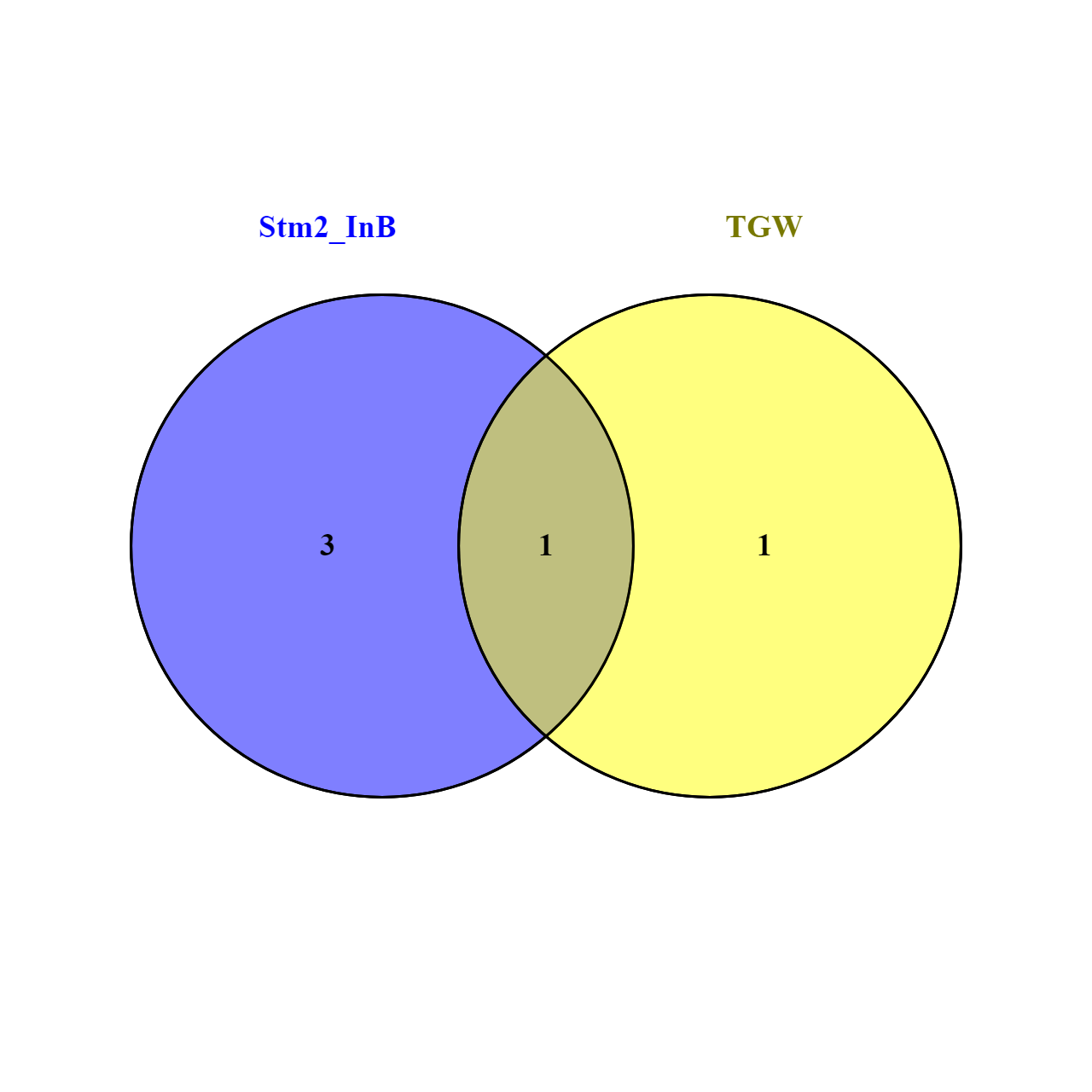

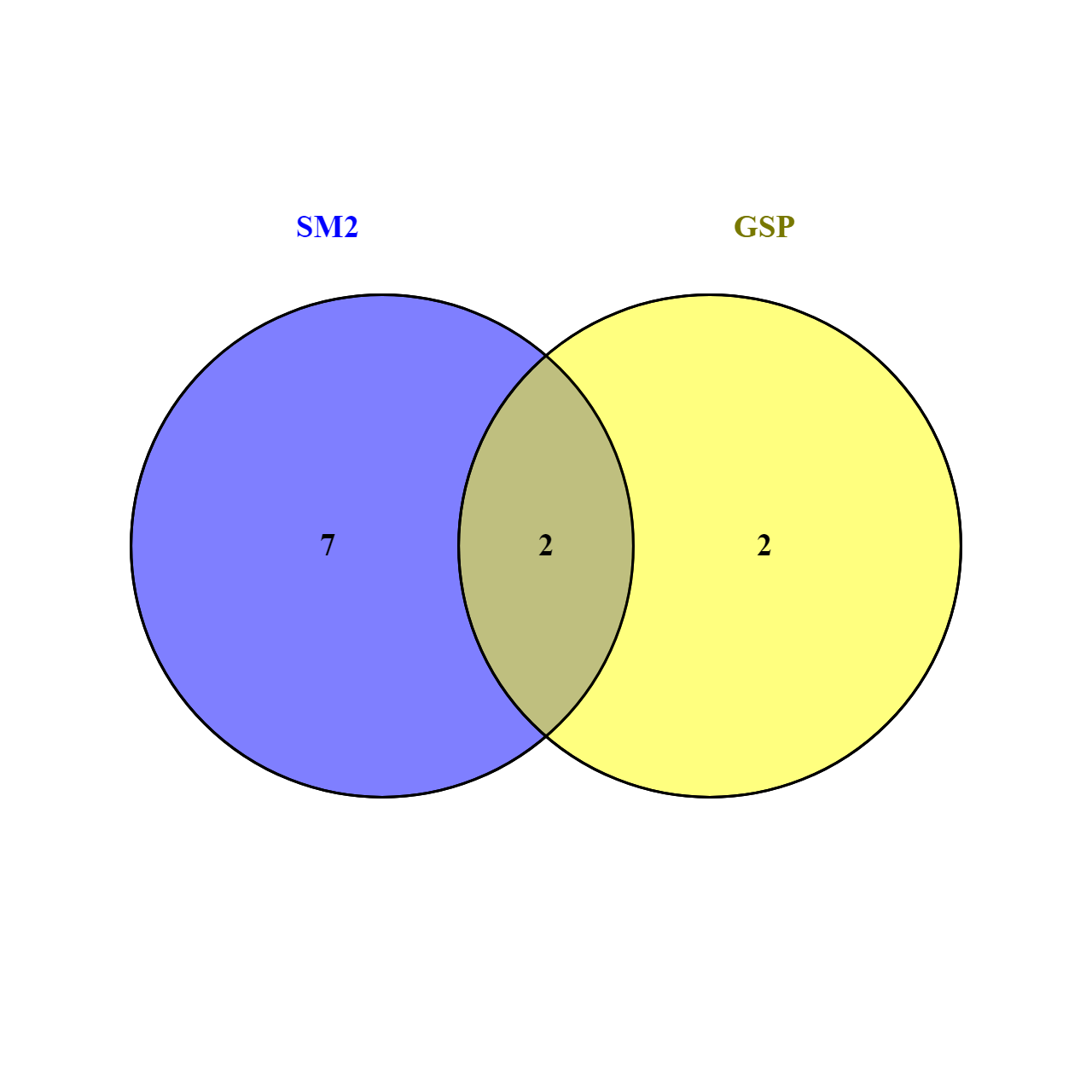

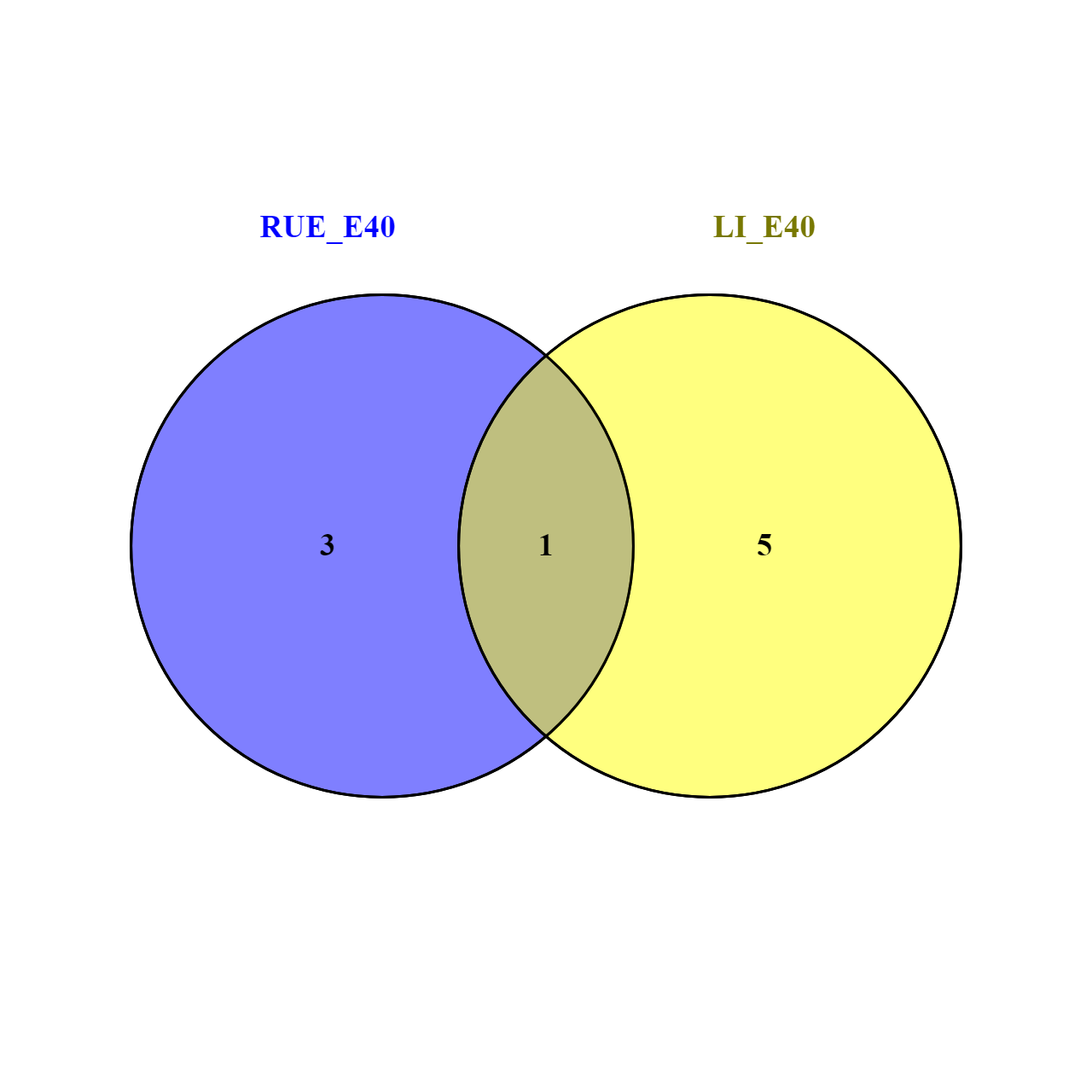


**b**

**e**

**d**

**c**

**a**

**Figure S5**. Venn diagram exhibiting the number of total and common MTA’s detected for (a) DTA, DTInB, PGF and SPKLSP^-1^, (b) RUET, RUE_GF, BM_PM and Yield, (c) Stm2_InB and TGW, (d) SM2 and GSP and (e) RUE_E40 and LI_E40. Information about the markers detected for each trait is presented on Table S6**.** Venn diagrams were created used the online tool from Oliveros, J.C. (2007-2015) Venny. An interactive tool for comparing lists with Venn's diagrams. <http://bioinfogp.cnb.csic.es/tools/venny/index.html>.
